# Identification of pleiotropic loci mediating structural and non-structural carbohydrate accumulation within the sorghum bioenergy association panel using high-throughput markers

**DOI:** 10.3389/fpls.2024.1356619

**Published:** 2024-02-28

**Authors:** Neeraj Kumar, J. Lucas Boatwright, Richard E. Boyles, Zachary W. Brenton, Stephen Kresovich

**Affiliations:** ^1^ Advanced Plant Technology, Clemson University, Clemson, SC, United States; ^2^ Department of Plant and Environmental Sciences, Clemson University, Clemson, SC, United States; ^3^ Pee Dee Research and Education Center, Clemson University, Florence, SC, United States; ^4^ Carolina Seed Systems, Advanced Plant Technology, Clemson University, Clemson, SC, United States; ^5^ Feed the Future Innovation Lab for Crop Improvement, Cornell University, Ithaca, NY, United States

**Keywords:** bioenergy association panel, biomass and composition, structural and nonstructural carbohydrates, whole-genome sequencing, high-throughput markers

## Abstract

Molecular characterization of diverse germplasm can contribute to breeding programs by increasing genetic gain for sorghum [*Sorghum bicolor* (L.) Moench] improvement. Identifying novel marker-trait associations and candidate genes enriches the existing genomic resources and can improve bioenergy-related traits using genomic-assisted breeding. In the current scenario, identifying the genetic loci underlying biomass and carbon partitioning is vital for ongoing efforts to maximize each carbon sink’s yield for bioenergy production. Here, we have processed a high-density genomic marker (22 466 550) data based on whole-genome sequencing (WGS) using a set of 365 accessions from the bioenergy association panel (BAP), which includes ~19.7 million (19 744 726) single nucleotide polymorphism (SNPs) and 2.7 million (~2 721 824) insertion deletions (indels). A set of high-quality filtered SNP (~5.48 million) derived markers facilitated the assessment of population structure, genetic diversity, and genome-wide association studies (GWAS) for various traits related to biomass and its composition using the BAP. The phenotypic traits for GWAS included seed color (SC), plant height (PH), days to harvest (DTH), fresh weight (FW), dry weight (DW), brix content % (BRX), neutral detergent fiber (NDF), acid detergent fiber (ADF), non-fibrous carbohydrate (NFC), and lignin content. Several novel loci and candidate genes were identified for bioenergy-related traits, and some well-characterized genes for plant height (*Dw1* and *Dw2*) and the *YELLOW SEED1* locus (*Y1*) were validated. We further performed a multi-variate adaptive shrinkage analysis to identify pleiotropic QTL, which resulted in several shared marker-trait associations among bioenergy and compositional traits. Significant marker-trait associations with pleiotropic effects can be used to develop molecular markers for trait improvement using a marker-assisted breeding approach. Significant nucleotide diversity and heterozygosity were observed between photoperiod-sensitive and insensitive individuals of the panel. This diverse bioenergy panel with genomic resources will provide an excellent opportunity for further genetic studies, including selecting parental lines for superior hybrid development to improve biomass-related traits in sorghum.

## Introduction

Sorghum [*Sorghum bicolor* (L.) Moench] is a multipurpose crop used as a significant source of food, feed, and bioenergy production. It is among the five most widely cultivated cereals worldwide, including wheat (*Triticum aestivum* L.), maize (*Zea mays* L.), rice (*Oryza sativa* L.), and barley (*Hordeum vulgare* L.) (FAOSTAT, 2021). Sorghum originated in the northeast of Africa circa 3000 B.C.E., most likely in the Sahel region, where it is one of the most important cereal crops due to its drought tolerance (Kebede, 1991; Ayana and Bekele, 1998). Moreover, sorghum is a versatile crop adapted to diverse soil and climate conditions, making it a promising alternative for energy production worldwide (Ahmad Dar et al., 2018). Sorghum is grown in various environments worldwide, including temperate and tropical regions. The photoperiod-sensitivity has been extensively studied in sorghum, which is regulated by at least six maturity genes, *Ma1*-*Ma6* (Quinby, 1967; Major et al., 1990; Rooney and Aydin, 1999).

The established sorghum racial structure is essentially a product of the broad geographic distribution, independent domestication events, and years of selection for advantageous traits in sorghum across those diverse atmospheres (Morris et al., 2013). These processes have resulted in distinguishing five botanical races, including bicolor, caudatum, durra, guinea, and kafir, which collectively demonstrate broad genotypic and phenotypic variation, particularly across panicle architecture and seed characteristics. Sorghum is a C_4_ grass that exhibits significant divergence in carbon partitioning across diverse subpopulations. Among the other plant sources exploited as feedstocks, sorghum efficiently accumulates high biomass with minimal inputs. The phenotypic variation present across sorghum has also permitted the classification of individual accessions based on differences in carbon partitioning (Boatwright et al., 2021). The carbon partitioning in sorghum contributes to four primary sink types: (1) cellulosic, where carbon is accumulated as C_5_ sugars primarily in the stem; (2) forage, where accumulation prioritizes the leaf volume; (3) grain, which stores carbon as starch in the grain; and (4) sweet, where carbon is stored in the stem as non-structural (C_6_) sugars (i.e., fructose, glucose, and sucrose). Identifying the genetic loci underlying carbon partitioning is vital for ongoing efforts to maximize each carbon sink’s yield. As the maximization of carbon accumulation is not a zero-sum process among the carbon types, knowledge obtained concerning one carbon partitioning process may improve the production in another.

Genome-wide association studies (GWAS) identify the association between molecular markers and quantitative traits (Zhu et al., 2008). Single nucleotide polymorphisms (SNPs) associated with a variety of phenotypic traits have been identified through GWAS using landraces, diverse accessions, or genetic mapping populations of sorghum, including traits related to plant architecture (Morris et al., 2013; Zhao et al., 2016; Hu et al., 2019; Kumar et al., 2023), agronomy (Rhodes et al., 2014; Boyles et al., 2017; Chopra et al., 2017; Li et al., 2018; Hu et al., 2019; Boatwright et al., 2022a; Kumar et al., 2023), bioenergy (Murray et al., 2008a and Murray et al., 2009; Brenton et al., 2016; Boyles et al., 2017, 2019; Souza et al., 2021), and biomass and its compositional traits (Brenton et al., 2016; Boatwright et al., 2022b).

Despite existing research, genetic improvement of sorghum for bioenergy is still challenging due to an incomplete understanding of the genetic architecture of the most relevant bioenergy traits. The selection for biomass and bioenergy-producing sorghum cultivars/hybrids depends on the characterization of biomass-related traits (*e.g*., plant height, fresh biomass yield, dry biomass yield, and sugar content). Early studies of genetic diversity in sorghum were conducted using association mapping panels and biparental populations for various traits, including agronomic (Casa et al., 2008) and bioenergy-feedstock (Murray et al., 2008a **;** Murray et al., 2009). Later, a substantial genetic resource was constructed, which was coined the bioenergy association panel (BAP), to study biomass-related traits to capture most of the sorghum feedstock sustainable to produce bioenergy and renewable chemicals (Brenton et al., 2016). The BAP comprised 238 high-biomass sorghum and 152 sweet sorghum accessions from the National Plant Germplasm System (NPGS). Features of various accessions within the BAP included high stalk height, photoperiod sensitivity, late maturing, and anthracnose resistance (Brenton et al., 2016). Plant height facilitates fresh and dry weight yield, essential breeding targets for bioenergy feedstock, forage, and cellulosic traits. The genetic characterization of the BAP was initially conducted using marker sets (232,303 SNPs) derived using genotyping-by-sequencing (GBS). This initial genetic study investigated the genomic differences between the biomass and sweet sorghum types (Brenton et al., 2016). In addition, several studies were also conducted based on GWAS using both the BAP and the sorghum association panel (SAP) where several major loci were determined to be associated with various important traits (Murray et al., 2008b; Shiringani and Friedt, 2011; Morris et al., 2013; Hayes, 2015; Cuevas et al., 2018; Li et al., 2018; Cuevas et al., 2019; Punnuri et al., 2022). Recently, a multi-parent population was developed using diverse parents from the BAP. This carbon-partitioning nested-association mapping (CP-NAM) panel was characterized for agronomic, biomass yield, and biomass-compositional traits (Boatwright et al., 2022b). However, these studies dealt with either low marker coverage or a few diverse lines compared to the current study, where several million genome-wide markers were identified.

In the present study, we used whole-genome high-throughput markers to characterize the bioenergy association panels for various biomass and compositional traits. Our objectives were (i) to study the genetic diversity, including nucleotide diversity, relative heterozygosity, and linkage disequilibrium of the panel (ii) to identify significant marker-trait associations for biomass yield and biomass composition (structural and non-structural carbohydrates), (iii) to identify significant loci with pleiotropic effects for multiple traits related to biomass yield (DTH, PH, FW, and DW) and its composition (NDF, ADF, NFC, and lignin) (iv) to develop genetic and genomic resources for sorghum research community.

## Materials and methods

### Plant materials

The details of the whole bioenergy association panel (BAP) and the plant introduction (PI) numbers are fully described by Brenton et al. (2016). The seed can be requested via the USDA Germplasm Repository Information Network (GRIN) using the PI number. The BAP contains 390 accessions, and it includes all five major sorghum races (bicolor, caudatum, durra, guinea, and kafir), which represent the entire African continent, Asia, and the Americas (**Supplementary Table S1**). The whole panel can be classified broadly into two major sorghum types (sweet and biomass). In total, 152 accessions exhibit a Brix (BRX) content over 10% at the development stage or physiological maturity and are considered sweet lines, as previously defined as the sweet sorghum association panel (Murray et al., 2009) as well as the U.S. historic sweet sorghum panel (Wang et al., 2009). The biomass lines were selected based on the diversity of worldwide geographic distribution, racial categorization, and agronomic characteristics (Brenton et al., 2016). As part of the TERRA-REF project (http://terraref.org/), all the samples were shotgun sequenced (150-bp paired-end) on an Illumina X10 instrument at the HudsonAlpha Institute for Biotechnology (Songsomboon et al., 2021). Each sample was multiplexed and ran on a total of 123 lanes, resulting in an average of 30X coverage per sample. The raw sequencing reads are available through the TERRAREF project page of the CyVerse repository (http://datacommons. cyverse.org/browse/iplant/home/shared/terraref).

### Field evaluation and phenotypic analysis

Phenotypic data for all the traits used in the genome-wide association analysis was derived from the previously published dataset (Brenton et al., 2016). The field evaluation and trait phenotyping are summarized in the following section. The phenotypic data for the traits were also summarized in **Supplementary Table S1**. The field experiment was conducted in Florence, SC, at the Pee Dee Research and Education Center of Clemson University during the summers of 2013 and 2014. Details of seed preparations and seed treatment to control the weeds are given by Brenton et al. (2016). The BAP panel (390 accessions) was planted using the complete randomized block design (CRBD) with yearly replications. The field trials were planted on 0.76 m rows at a planting density of ~96,000 plants ha^1^ in loamy-sand soil on 16 May 2013 and 6 May 2014. The trials were irrigated at the time of planting and as needed. The field trials were not irrigated ~90 days after planting because some BAP accessions were taller than the irrigation pivot.

The measurements on plant height were taken at the stage of physiological maturity, or a set harvest date of October 1 of each year from the base of the stalk to the apex of the panicle or the apex of the shoot apical meristem if the panicle was absent (**Supplementary Table S1**). Fresh weight (FW) and dry weight (DW) were recorded, excluding the panicles and leaves of a stalk. FW was recorded based on the total weight of three harvested plants (~0.5 m of row length) from the base at the physiological maturity stage, excluding panicles. Most plots were harvested at the physiological maturity stage except for genotypes that did not flower, which were harvested at a single time. Before collecting dry weight data, each fresh stalk was dried at 40°C until a constant moisture content was obtained. The DW trait in tons ha^1^ was extrapolated based on the planting density of ~96,000 plants ha^1^. Each stalk sample was ground with a Retsch SM 300 cutting mill to estimate the biomass compositional parameters using a PerkinElmer DA7250TM NIR instrument (https://www.perkinelmer.com). Four biomass compositional parameters were measured, including acid detergent fiber (ADF), neutral detergent fiber (NDF), nonfibrous carbohydrates (NFC), and lignin. The NIR instrument uses the calibration curves for spectral measurements built using wet chemistry values generated by Dairyland Laboratories, Inc. (Arcadia, WI, USA), as described in Brenton et al. (2016). All compositional data are presented as a percentage of dry matter (DM). Mean values of each trait were used to perform all the phenotypic data analysis (correlation coefficient) and GWAS. Pearson’s correlation coefficient was estimated using the *metan* package and the *corr_plot* and *plot.corr_coef* functions were used to visualize correlation matrices for each trait in R software version 4.1.3 (Team RC, 2022).

### Genomic data processing

In this study, the sequencing data of 365 accessions of BAP were used. The accessions were sequenced using shotgun sequencing (150-bp paired-end) on an Illumina X10 instrument at the HudsonAlpha Institute for Biotechnology as part of the TERRA-REF project (http://terraref.org/) The individual samples were multiplexed and run on a total of 123 lanes, resulting in an average of 30X coverage per sample (Songsomboon et al., 2021). The genomic reads ~22 466 550 were accessed from Songsomboon et al. (2021) before processing them following the Genome Analysis Toolkit (GATK) best practices pipeline version 4.1.7.0 (McKenna et al., 2010). Reads were filtered using fastp (Chen et al., 2018) and aligned to the third version of the sorghum BTx623 reference genome (McCormick et al., 2018) using Burrows-Wheeler aligner (BWA) version 0.7.17 (Li and Durbin, 2010). The resulting sequence alignment and map (SAM) files were sorted and converted to the binary alignment and map (BAM) using samtools version 1.9 (Danecek et al., 2021) before marking duplicates using the MarkDuplicates command in GATK version 4.2.6.1 (DePristo et al., 2011; Van der Auwera et al., 2013). BAM files were then recalibrated using the BaseRecalibrator and ApplyBQSR commands in GATK using the quality-filtered reads from the BAP with MAF > 0.05 (http://terraref.org/). The GATK HaplotypeCaller was then used to generate genome variant call format (gVCF) files from individual samples before importing the genotypic data from each chromosome into joint calling databases using GenomicsDBImport. Joint calling (GenotypeGVCFs in GATK) was performed on chromosome halves (split on centromeres) to parallelize better variant calling. SNPs were filtered stringently for quality (QD < 2.0, InbreedingCoeff< 0.0, QUAL< 30.0, SOR > 3.0, FS > 60.0, MQ < 40.0, MQRankSum< −12.5, and ReadPosRankSum< −8.0), missing data (50%), and minor allele frequency (MAF > 0.05) using both GATK and BCFtools (version 1.11), which are the programs for variant calling and manipulating files in the Variant Call Format (VCF) in a binary manner before performing GWAS (Danecek et al., 2021). Beagle (version 5.3) was used to impute missing genotype data in the VCF file assembled from the GATK pipeline (Browning et al., 2021).

### Population diversity and structure

Population structure was estimated from the pruned SNPs using ADMIXTURE version 1.3.0 (Alexander and Lange, 2011) to identify subpopulations (*K*) in the BAP. ADMIXTURE is a highly efficient tool and easy to use for ancestry estimation from SNP datasets. Variants were filtered using a minor allele frequency (MAF > 0.05) using the R package (version 4.1.3; Team RC, 2022). The filtered variants were used in ADMIXTURE to estimate the population structure. Five-fold cross-validation was used to determine the optimal number of ancestral populations, *K*, by selecting the model with the lowest cross-validation error (*K*=8). The Q matrix of the selected model – representing the ancestry fractions of individuals was then sorted by ancestry coefficient for each subpopulation such that individuals with coefficients > 50% were assigned to the corresponding subpopulation. Subpopulations were classified as *K1*-*K8* as determined by the sorted ancestry coefficient column. Principal component analysis (PCA) was performed using the 5.48 million SNPs to determine the optimum number of clusters using a complete BAP set and assess the genomic variation captured by each PC. The filtered variants (MAF > 0.05) were used for estimating principal components (PCs) using the GAPIT package in R (version 4.1.3; Team RC, 2022). This classification was used to represent the ancestral admixture of individuals in PCA of the BAP. The inbreeding coefficient and nucleotide diversity were calculated using VCFtools version 0.1.16 (Danecek et al., 2011). Genome-wide *F_st_* were estimated using bcftools (Danecek et al., 2021). A window size of 1 Mb with a step size of 100 kb was used for calculation. *F_st_* estimates were calculated for each subpopulation against all other subpopulations, and the mean *F_st_* for a subpopulation at a genomic window was computed as the average *F_st_* of a subpopulation against all other subpopulations for that genomic window. We also computed *F_st_* between accessions derived from the sorghum photoperiod-sensitive and photoperiod-insensitive accessions within the BAP using the same parameters mentioned above (**Supplementary Table S2**). Tajima’s D for the whole panel was calculated for 1-Mb non-overlapping windows using the vcftools function –TajD.

### Genome-wide association studies

The phenotypic data collected on BAP were used by Brenton et al. (2016) for performing GWAS. We performed GWAS to identify significant marker-trait associations using a Memory-efficient, Visualization-enhanced, and Parallel-accelerated (*rMVP*) program (Yin et al., 2021) installed in the R version 4.1.3. programming language (Team RC, 2022). The program ‘*rMVP’* was designed to perform GWAS more efficiently for large datasets. The *rMVP* is an efficient program for evaluating the population structure and implementing parallel-accelerated association tests to improve overall computation time dramatically. This study used two popular models; the mixed linear model (MLM; Zhang et al., 2010a) and the fixed and random model circulating probability unification (FarmCPU; Liu et al., 2016). The MLM warrants a single-locus analysis, where individuals are included as random effects, and the degree of correlation among individuals is determined using a kinship (K) matrix. The use of the MLM further provides shrinkage to the model such that potential false positives due to shared ancestry are no longer significant. An MLM can be described using Henderson’s matrix notation (Kumar et al., 2023) as follows:


(1) Y=Xβ+Zu+e

where Y is the vector of observed phenotypes; β is an unknown vector containing fixed effects, including the genetic marker, population structure (Q), and the intercept; u is an unknown vector of random additive genetic effects for individuals; X and Z are the known design matrices for fixed and random effects, respectively; and e is the unobserved vector of residuals. The u and e vectors are assumed to be normally distributed with zero mean and unit variance.

FarmCPU is a multi-locus model that uses recurrent fixed and random effect models to generate sets of pseudo-quantitative trait nucleotides (QTNs) to use as covariates and controls for false positives during analysis (Liu et al., 2016). Comparatively, FarmCPU is a more efficient model as it removes confounding between kinship and the testing marker. By iterating a fixed effect model to identify significant pseudo-QTNs to use as covariates in a random effect model using a restricted kinship matrix like the SUPER algorithm (Wang et al., 2014) to further refine the set of included covariates by maximizing the likelihood of the random effects model. Iterations cease when no change occurs in the estimated set of pseudo-QTNs. The criterion for each significant marker-trait association corresponding to putative SNPs was based on the Bonferroni-corrected *p*-value threshold 9.1e^-9^. The above threshold was calculated using 0.05/*m*, with *m* being the number of markers at 5.48 million SNPs.

### Pleiotropic effects

To assess the significant pleiotropic effects of loci on the set of BAP traits (SC, DTH, PH, FW, DW, BRX, ADF, NDF, NFC, and lignin), a multivariate adaptive shrinkage approach was used following the *mashr* model in R (Urbut et al., 2019). The effect sizes and standard errors for every SNP marker in the MLM and FarmCPU for the above traits were filtered using a local false sign rate (LFSR)< 0.1 based on a condition-by-condition analysis using *mashr* in R (Stephens, 2017). Here, LFSR represents the probability of incorrectly assigning the direction of an effect. The LFSR provides a superior measure of significance over traditional multiple-testing corrections such as Bonferroni or False Discovery Rate (Benjamini and Hochberg, 1995) due to its robust estimation process (Stephens, 2017). A control set of estimated effects and standard errors was also randomly selected from the 5.3 million markers to estimate the covariance between the markers for each phenotype. Using this control set, a correlation matrix was estimated using *mashr* (Urbut et al., 2019) to control for any confounding effects arising from correlated traits. The pleiotropic effects across traits were tested using canonical and data-driven covariance matrices. The posterior probabilities were estimated for each SNP by fitting a *mash* model on all tests. Bayes factors were extracted, and a Manhattan plot was generated from *mash* results using the CDBN genomics R package (MacQueen et al., 2020). The variants exhibiting Bayes Factors > 3 represented significant pleiotropic effects.

## Results

### Phenotypic trait relationship and heritability

Previously, the phenotypic diversity, heritabilities, and correlations among the traits have been reported by Brenton et al. (2016). The relationship between phenotypic traits and heritabilities is summarized to recall these features (**Supplementary Table S3**). PH was positively correlated with DTH, FW, DW, ADF, NDF, and lignin but did not show a correlation with NFC and BRX. Similarly, DTH was also positively correlated with all the traits except NFC and BRX. FW showed a positive correlation with DW and a moderate to poor correlation with lignin, ADF, and NFC but no correlation was exhibited with NDF and BRX. However, DW showed a moderate correlation with BRX, NFC, and lignin, but no relationship was observed with ADF and NDF. The ADF and NDF showed a strong positive relationship but exhibited a negative correlation with NFC and BRX. Lignin was negatively correlated with NFC and BRX. However, the NFC showed a negative relationship with all the traits except BRX (**Supplementary Table S3**). The heritability estimates of broad sense were highest for PH and moderate for ADF, NDF, NFC, and lignin, though they were lowest for DW.

### Sequencing, population structure and linkage disequilibrium

As mentioned, the BAP was initially developed as a sorghum diversity panel and characterized using a set of GBS markers (232,303 SNPs) by Brenton et al. (2016). In this study, we used whole genome sequencing (WGS) data with 22 466 550 sequences, which include SNPs (~19 744 726) and ~2 721 824 insertions and deletions (indels) identified using the GATK analysis pipeline. Of these 19 744 726 SNPs, 5 485 810 (~5.48 million) high-quality filtered SNPs were identified and subsequently used for association mapping analysis after filtering for missing data (> 0.3) and minor allele frequency (MAF)< 0.05 (Zhou and Stephens, 2014). As for indels, a significant proportion (2.6 million) were small, and 44,459 were large with sizes ≥ 50 bp. The sequencing data showed higher SNP density on chromosome arms, specifically in telomeric regions instead of centromeric regions of the genome (**Supplementary Figure S1**). We observed a larger number of transitions (~13 million) compared to transversions (> 6 million) (**Supplementary Table S5**).

**Figure 1:**
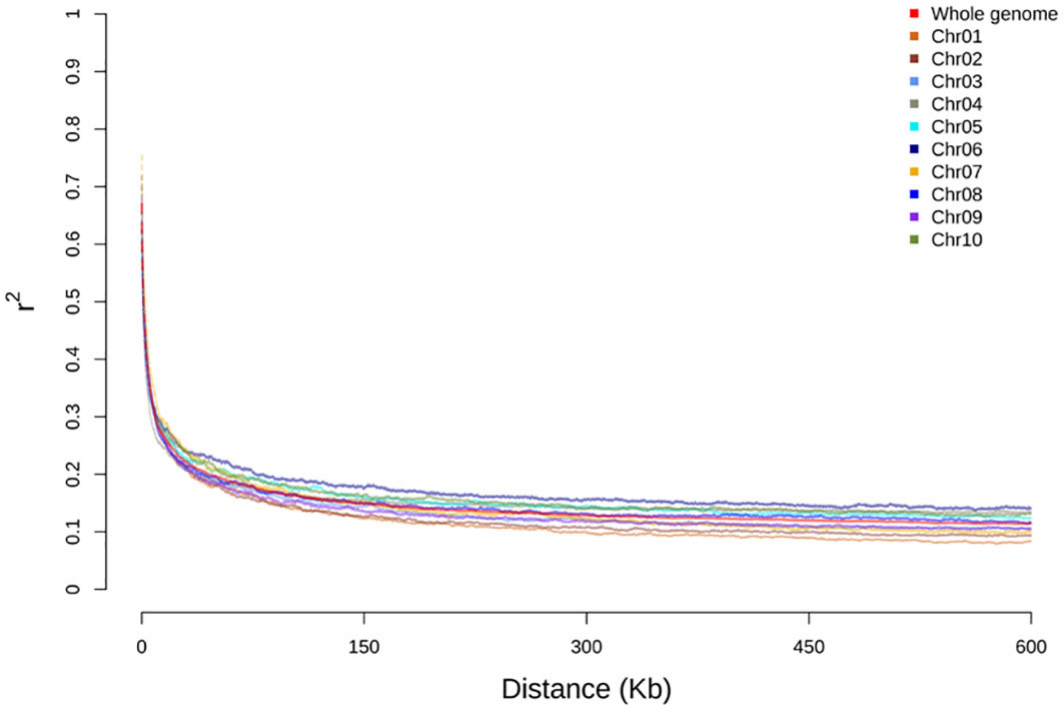
Linkage disequilibrium (LD) decay (Pearson’s correlation coefficient squared) of the bioenergy association panel (BAP) population plotted against the distance in kilobase (kb) chromosome-wise and across the genome.

The linkage disequilibrium (LD) impacts the haplotype construction and genetic mapping resolution. In this study, LD analysis was performed to assess the distances and pattern of LD decay for individual chromosomes and genome-wide in the BAP. The coefficient of determination (*r^2^*) between markers on each chromosome was also measured to estimate the LD relationship between genomic loci. The LD decay plots for each chromosome and genome-wide were created by plotting *r^2^* on the y-axis and physical distances in kilobase (kb) on the x-axis. The genome-wide average LD for the BAP fell around *r^2^* < 0.2 after 40 kb, and the LD decay leveled out around 160 kb (**Figure 1**). The average LD decay for individual chromosomes ranged (~ 30-80 kb). This panel exhibited lower LD throughout the chromosomes and genome-wide compared to the grain sorghum panel (SAP) of grain sorghum (Boatwright et al., 2022a). However, chromosome 6 of the BAP exhibited a slightly higher LD that fell around *r^2^* < 0.2 after ~80 kb, slightly higher than the overall genome-wide LD (**Figure 1**).

**Figure 2:**
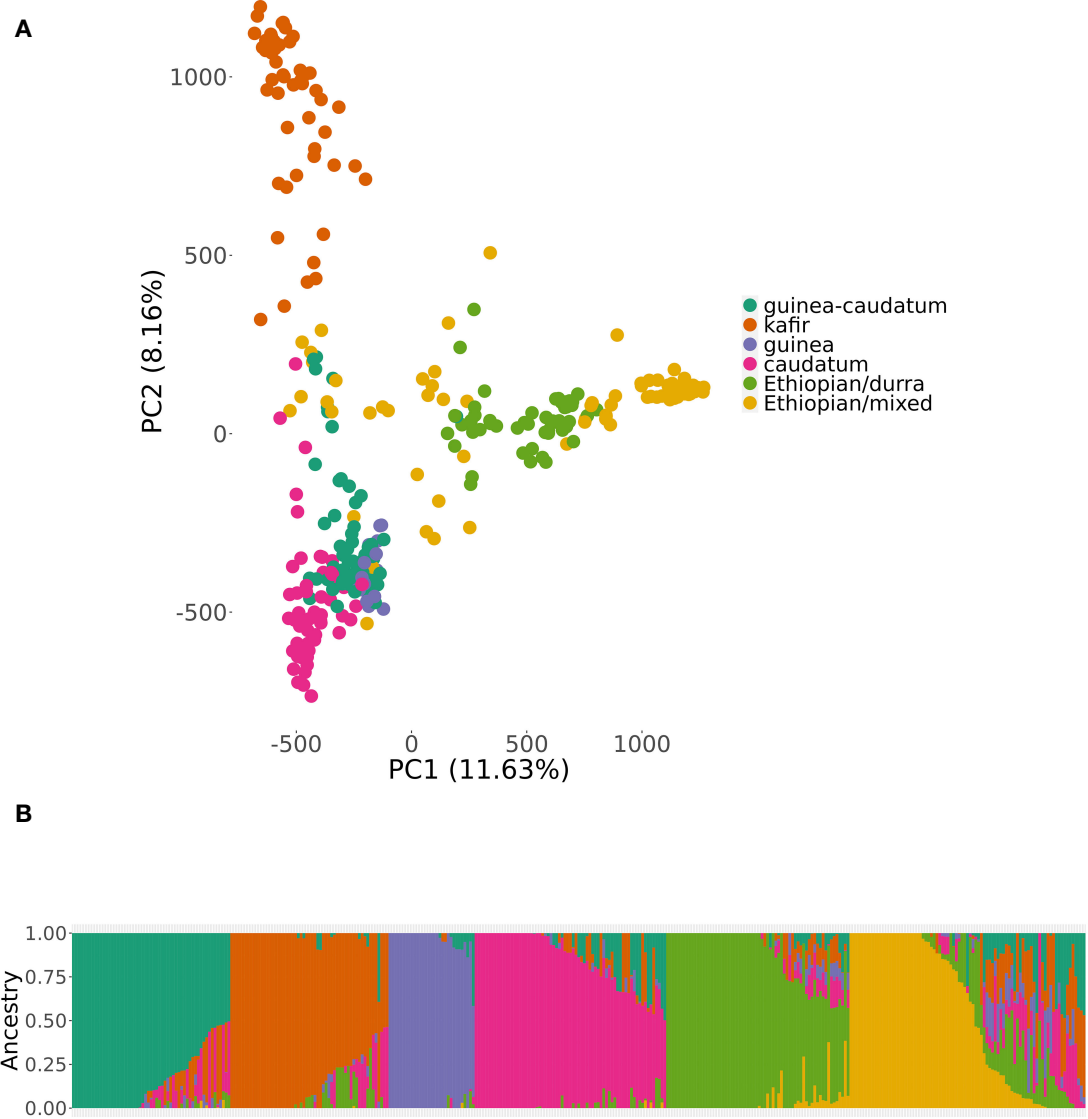
Population structure within the bioenergy association panel (BAP) using principal component analysis (PCA) and an admixture model (*K* = 6). Subpopulations were labeled with corresponding botanical races or sorghum types that predominated for a given subpopulation. Subplots represent the **(A)** projection of BAP accessions by the principal component (PC1) and PC2. **(B)** the degree of admixture across the subpopulations using consistent subpopulation colors across all subplots. The color represents the racial grouping of BAP; red = Guinea-Caudatum, blue = Kafir, green = Guinea, purple = Caudatum, orange = Ethiopian-Durra, and yellow = Ethiopian-mixed.

The results of genomic variations explained through PCA using the BAP and the SNP data (5.48 million) are demonstrated in **Figure 2A**. The PCA results assist in the estimating and visualizing of genetic relatedness across the accessions and further describes the population stratification in the BAP. First two PCs accounted for 19.79% (PC1: 11.63%, PC2: 8.16%), a significant proportion of the genomic variation. The optimal *K* value was confirmed using 365 accessions of BAP with lower Bayesian information criterion (BIC) following ADMIXTURE analysis (**Figure 2B**). The subpopulation grouping in the population structure analysis led to six clusters, including three that correspond to the three botanical races of sorghum (kafir, guinea, and caudate). The smallest group was guinea (31 accessions), kafir (57 accessions) and caudatum (69 accessions). The fourth and fifth subpopulations comprised guinea-caudatum (57) and Ethiopian-durra (66). Last, the sixth subpopulation comprised the most prominent group (85 accessions) of the Ethiopian-mixed race. The fifth and sixth sub-populations mainly comprised a group of mixed-racial and Ethiopian-mixed, and the accessions were not classified as bicolor.

Eight genomic regions showed selection sweeps between photoperiod-sensitive and insensitive accessions using *F_st_* estimates (**Supplementary Figure S2A**). Of these eight, six genomic regions showed strong selection sweeps on six different chromosomes (Chr1, Chr2, Chr3, Chr5, Chr6, and Chr8), including two minor peaks on Chr4 and Chr10 (**Supplementary Figure S2A**). The most substantial sweeps were observed around 67-68 Mb on Chr1 and 40-43 Mb on Chr6 (**Supplementary Figure S2A**). The genomic region on Chr1 harbors the yellow seed color locus (*Y1:* Sobic.001G398100). However, a genomic region of Chr6 contains a plant height locus (*Dw2*: Sobic.006G067700). Additional peaks were observed on Chr2 (~ 2 Mb), Chr3 (17-20 Mb), Chr5 (35 and 59 Mb), and Chr8 (5-6 Mb). We also calculated the expected heterozygosity (2pq) on a per-site basis using allele frequencies (p and q) for the photoperiod-sensitive and insensitive group of accessions (**Supplementary Figure S2B**). Genetic variation in relative heterozygosity between the two groups (photoperiod-sensitive and insensitive accessions) was consistent and in agreement with the distribution of *F_st_* peaks between the two groups. Tajima’s D test assessed the nucleotide diversity between the BAP groups (**Supplementary Figure S2C**). The whole genome average value for Tajima’s D was 3.01, indicating the small number of rare alleles because of extensive inbreeding. Most of the genomic regions showed Tajima’s D above the mean value, indicating balancing selection, while some regions, particularly at Chr4, Chr7, and Chr9, showed bottlenecks indicative of purifying selection (**Supplementary Figure S2C**). Some regions in the middle of Chr2 and Chr7 showed a substantial bottleneck in Tajima’s D and expected heterozygosity for photoperiod-sensitive lines compared to the insensitive lines (**Supplementary Figure S2B**).

### GWAS and candidate gene identification for biomass-related traits

**Figure 3:**
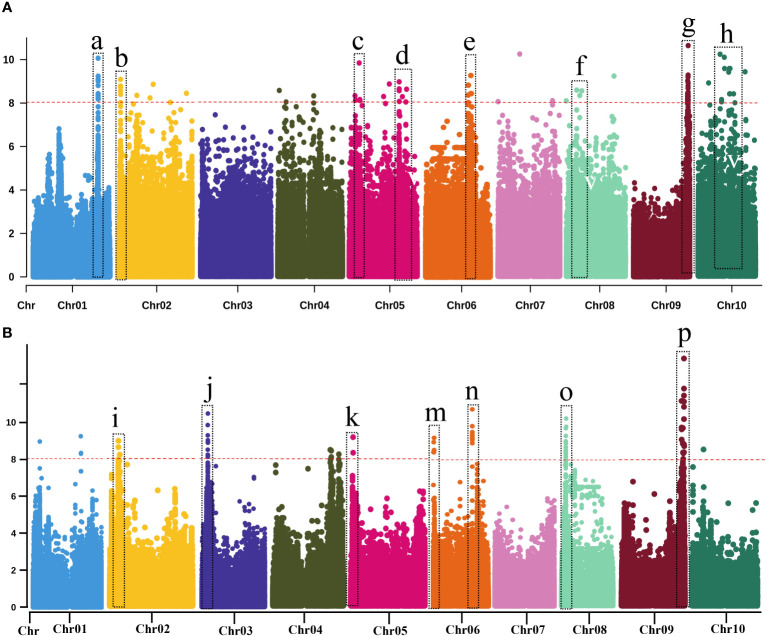
Manhattan plots of genome-wide association using MLM model with highly significant genes or loci of various traits. Vertical dotted bars show genes and loci **(A)** related to agronomic and biomass yield-related traits and **(B)** biomass compositional traits. (a) Seed color (b–d, h) Dry weight (e, g) for PH (f) Fresh weight (I, k, p) for NDF (j, m, n) for ADF (o) for NFC. The -log_10_ (*p*) values (*y*-axis) are plotted against the position on each chromosome (*x*-axis). Each solid circle represents a SNP, and the red dashed line represents the Bonferroni-corrected threshold (*p* ≤ *0.05*).

Seed color is a highly heritable and well-characterized trait in sorghum. We performed GWAS analysis for seed color to validate our genomic data in BAP (**Table 1**; **Supplementary Table S7**; **Figure 3A**-GWAS; **Supplementary Figure S3**). In total, nine significant loci associated with seed color phenotypes were identified on seven chromosomes (Chr1, Chr2, Chr4, Chr6, Chr7, Chr8, and Chr10). A highly significant locus was identified on Chr1 (Chr01: 68,401,502), which corresponded to Sobic.001G398100 was recently confirmed the location of *YELLOW SEED1* (*Y1*) locus (Nida et al., 2019; Boatwright et al., 2022a). The *Y1* gene was also confirmed by a previous study by Brenton et al. (2016) using the BAP. Another highly significant locus was identified on Chr6 (~42 Mb) near the *Dw2* locus. Two significant loci associated with seed color were identified on Chr4 (54,654,878 and 58,220,148 bp). Several novel loci were identified on Chr2 (53 and 62 Mb), Chr4 (54 and 58 Mb), Chr7 (62 Mb), Chr8 (13 Mb), and Chr10 (60 Mb).

**Table 1:** A summary of highly significant associations identified for various traits using BAP.

Trait*	Chromosome	Position (bp)	Effect	SE	Probability
SC	Chr01	68,401,502	0.20	0.03	9.3E-11
	Chr02	62,085,143	0.20	0.02	7.7E-18
PH	Chr06	42,867,057	-88.98	14.67	8.6E-10
	Chr07	2,812,493	17.92	3.62	8.5E-09
	Chr09	57,040,002	69.69	11.73	7.4E-09
DTH	Chr01	17,500,538	3.59	0.49	9.1E-16
	Chr04	27,725,942	-5.27	0.96	9.1E-13
	Chr06	41,098,789	-10.78	1.83	9.0E-09
	Chr06	1,837,917	4.43	0.53	7.85E-10
	Chr09	58,757,856	3.49	0.65	7.6E-10
FW	Chr02	77,472,104	0.89	0.14	9.8E-10
	Chr05	52,717,775	0.99	0.16	7.2E-10
	Chr06	48,272,492	-0.43	0.07	6.5E-12
	Chr08	40,365,266	-0.68	0.11	8.3E-09
DW	Chr01	32,303,678	0.35	0.05	9.3E-11
	Chr01	48,608,225	-0.25	0.04	8.7E-10
	Chr02	2,178,092	0.18	0.03	8.1E-10
	Chr02	77,472,104	0.89	0.14	9.8E-10
	Chr04	8,971,818	0.31	0.05	9.1E-09
	Chr05	6,633,276	0.19	0.03	7.5E-09
	Chr05	52,831,342	0.19	0.03	8.6E-09
	Chr05	59,785,398	0.17	0.03	9.1E-09
	Chr07	179967	0.33	0.06	8.4E-09
	Chr07	60,434,191	0.08	0.01	9.0E-14
	Chr08	8698	0.16	0.03	7.7E-09
	Chr10	25,056,054	0.20	0.03	8.1E-09
	Chr10	39,186,221	0.28	0.05	9.0E-09
BRX	Chr06	43,928,936	1.63	0.27	9.4E-10
ADF	Chr01	56,251,699	-5.66	0.94	7.4E-10
	Chr03	7,385,645	-6.09	0.96	9.4E-10
	Chr06	878481	-6.03	0.96	9.8E-10
	Chr08	630891	-5.30	0.90	9.0E-09
NDF	Chr01	24,419,224	-26.80	4.41	6.0E-10
	Chr01	67,432,697	-8.66	1.47	9.0E-09
	Chr02	8,215,290	-11.09	1.76	9.8E-10
	Chr02	5,362,3849	-8.45	1.44	9.0E-11
	Chr04	56,527,960	-12.77	2.16	8.0E-09
	Chr05	2,240,342	-15.54	2.44	6.0E-10
	Chr06	60,028,378	-11.97	1.99	4.6E-09
	Chr08	10,794,424	12.54	1.99	9.1E-10
	Chr08	17,452,222	-16.67	2.24	8.6E-13
	Chr08	18,159,519	-16.67	2.24	8.6E-13
	Chr08	31,808,548	-14.48	2.30	9.4E-10
	Chr08	38,151,176	-16.57	2.23	1.0E-12
	Chr08	40,563,245	-14.48	2.30	9.4E-10
	Chr08	44,335,003	-16.11	2.28	9.8E-12
	Chr08	45,221,686	-15.99	2.26	9.9E-12
	Chr08	49,395,916	-14.39	2.14	8.6E-11
	Chr08	50,533,766	-13.43	2.25	6.2E-09
	Chr09	58,569,152	-18.67	2.34	8.5E-19
	Chr10	10,656,212	-9.89	1.61	2.5E-09
NFC	Chr03	12,740,909	-2.20	0.44	7.6E-09
	Chr03	18,556,009	6.74	1.13	6.3E-09
	Chr03	20,130,207	7.27	1.15	8.6E-10
	Chr05	35,131,508	13.07	2.19	6.6E-09
	Chr06	43,346,363	6.74	1.14	8.5E-09
	Chr06	48,838,810	9.21	1.37	9.8E-11
	Chr06	50,242,536	-1.51	0.28	7.5E-10
	Chr08	5,603,293	7.60	1.29	9.1E-09

SC, seed color, PH, plant height, DTH, days to harvest, FW, fresh weight, DW, dry weight, BRX, brix %, ADF, acid detergent fiber, NDF, neutral detergent fiber, NFC, non-fibrous carbohydrate. SE, standard error.

We performed GWAS for PH, which is an important trait in sorghum, irrespective of end-use. Nine loci were identified for PH on seven different chromosomes (Chr1, Chr4, Chr6, Chr7, Chr8, Chr9, and Chr10). A highly significant locus was identified on Chr9 (57,040,002 bp) corresponding to Sobic.009G229800. The functional annotation analysis confirmed that Sobic.009G229800 corresponded to *Dw1* (Brown et al., 2008; Klein et al., 2008). Two loci associated with PH were identified on Chr6 (12 and 43 Mb) were identified involving a locus at 43 Mb that corresponded to Sobic.006G071628, which was located within 60 kb of the *Dw2* (Sobic.006G067700; Higgins et al., 2014; Burrell et al., 2015). Two significant genetic loci were identified on Chr10 (1.8 and 12 Mb), including SNP (Chr10:1,841,997) located within 100 kb of the *waxy* locus (Sobic.010G022600), though seemingly unrelated to biomass. The rest of the four relatively minor genomic regions associated with PH were identified on Chr1 (20,134,329 bp), Chr4 (5,182,079), Chr7 (2,812,493 bp), and Chr8 (888,245 bp).

Eight loci were identified for DTH on five different chromosomes (Chr1, Chr4, Chr6, Chr9, and Chr10). Of these eight loci, a highly significant locus (14 SNPs) was detected on Chr6, within a 60 kb region to the *Dw2* gene (Sobic.006G067700), that showed shared associations for PH and DTH. Another significant locus was detected on Chr9, near another important dwarfing gene, *Dw1* (Sobic.009G229800). A significant locus was also identified on the Chr6 at 1.8 Mb, close to a known maturity gene *Ma6* (Murphy et al., 2014). Two additional loci were identified on Chr10 (~49 Mb and ~54 Mb) for the first time and thus were considered novel.

In total, 11 loci (19 SNPs) were significantly associated with FW, located on seven different chromosomes (Chr1, Chr2, Chr5, Chr6, Chr7, Chr8, and Chr9). Of these seven associations, four loci showed highly significant associations located on Chr2, Chr5, Chr6, and Chr8. A single locus was identified on Chr2 (77 Mb), Chr6 (48 Mb), Chr7 (1.4 Mb), and Chr9 (59 Mb). Of these 11, two loci were identified on Chr 1 (32 and 65 Mb) and Chr5 (19 and 52 Mb). For FW, three loci were detected on Chr8 (0.6, 40, and 61 Mb). For DW, 43 loci (107 SNPs) were identified, located on all the ten sorghum chromosomes except Chr3. Of these 43 associations, 12 genomic loci were considered highly significant. Of these 12, three significant loci were detected on Chr5 (6, 52 and 59-60 Mb), Chr7 (0.1 and 60 Mb), and Chr10 (25 and 39 Mb). Additional associations were detected, which included two loci on Chr2 (21 and 71 Mb) and a single locus on Chr1 (48 Mb), Chr4 (~9 Mb), and Chr8 (8 Mb). The remaining loci, including a locus on Chr6 and three loci on Chr9) were minor. A highly significant locus on Chr2 (Chr2: 71718582) corresponding to a candidate gene (Sobic.002G353800), which encodes the homeodomain-leucine zipper (HD-Zip) transcription factor family that plays a vital role in plant development and morphogenesis as well as responses to biotic and abiotic stresses (Prom et al., 2021). Interestingly, only a single genomic region on Chr5 (52 Mb) was co-localized between these traits (FW and DW). Altogether, GWAS identified (3 SNPs) significantly associated with BRX, which were located on Chr3 (~62 Mb), Chr6 (~ 43 Mb), and Chr8 (~18 Mb). A sole SNP (Chr6: 43,928,936) overlapped with a dwarfing locus (*Dw2*) of these three associations. This genomic region at ~43 Mb was within 0.9 Mb of the dwarfing gene *Dw2* (Sobic.006G067700).

### GWAS and candidate gene identification for biomass compositions

GWAS identified several genomic regions strongly associated with phenotypic traits related to biomass composition, including structural (ADF, NDF, and lignin) and non-structural carbohydrates (NFC) using the BAP (**Table 1**; **Supplementary Table S4**, **S7** **;** **Figure 3B** **;** **Supplementary Figure S3**). GWAS identified 11 significant loci (74 SNPs) for ADF on six different chromosomes (Chr1, Chr3, Chr6, Chr8, and Chr10). Of these 11 loci, eight were considered highly significant. Of these eight, two loci on each chromosome were located on Chr1 (8 Mb and 56 Mb), Chr6 (8.7 Mb and 43 Mb), and Chr8 (0.6 and 18 Mb). A significant locus associated with ADF was located on Chr3 (73 Mb) and Chr10 (~11 Mb).

For NDF, at least 67 highly significant loci (80 SNPs) were identified, located on all ten sorghum chromosomes except Chr3. Maximum significant associations (65 SNPs) were detected on Chr8 (5 to 49 Mb). A significant locus was identified on Chr1 (Chr01:67432697) corresponding to Sobic.001G386700, related to homeobox (*WOX*) genes from a large gene family expressed explicitly in plants. The *WOX* genes are known to play essential roles in regulating the development of plant tissues and organs by determining cell fate (Zhang et al., 2010b). A locus was identified on Chr5 (Chr5:2240342), corresponding to Sobic.005G024800, that encoded the zinc-induced facilitator-like 1 (ZIFL) transporter proteins. The ZIFL proteins are known to play an important role in mobilizing essential micronutrients in rice (Ricachenevsky et al., 2011). A significant SNP on Chr9 (Chr09:58569186) was identified, corresponding to Sobic.009G250600 that encodes an F-box protein, and its role might, therefore, be related to protein degradation via the ubiquitin-proteasome pathway, with a wide variety of possible physiological and developmental effects in plants (Zhang et al., 2019). Another significant locus on Chr9 (Chr09: 57597290) corresponded to a candidate gene (Sobic.009G237900) that encoded a putative plastocyanin (β-sheet proteins) that plays a significant role in photosynthesis, which impacts the dry biomass yield (Habyarimana et al., 2020). Additional loci associated with NDF were identified, which included a locus on Chr1 (24 Mb), two loci on Chr2 (~8 Mb and 53 Mb), and a single locus each on Chr4 (55-63 Mb), Chr5 (2.2 Mb), Chr6 (~60 Mb), and Chr10 (~10 Mb). Of these associations, a locus each on Chr1 (24 Mb), Chr2 (~53 Mb) and Chr4 (55-63 Mb), was detected for the first time, which were considered novel. However, the rest of the loci were corroborated with earlier studies.

For NFC, GWAS identified 15 significant associations (88 SNPs) located on Chr1, Chr3, Chr5, Chr6, Chr8, and Chr9. The most prominent genomic region (~ 65 kb) showed significant associations (75 SNPs) for NFC on Chr8 (5-6 Mb) and displayed a broad peak. Another highly significant locus was identified each on Chr1 (4 Mb) and Chr9 (0.5 Mb). Additionally, three genetic loci were identified each on Chr3 (12, 17, and 20 Mb), Chr5 (4, 35, and 60 Mb), and Chr6 (43, 48, and 50 Mb). The genomic region on Chr6 (Chr06: 48,838,810) associated with NFC had a gene coding for cellulase enzymes, Sobic.006G122200. This gene hydrolyzes glycosidic bonds in complex carbohydrates, such as cellulose, a significant component of NDF (Brenton et al., 2016). Some shared associations were also observed on Chr8 (~5.5 Mb) between NDF and NFC, possibly due to inverse relations between these traits. Surprisingly, GWAS identified only a sole QTL (1 SNP) at ~62 Mb on Chr8 associated with lignin content.

### Pleiotropic analysis

In this analysis, all the SNP effects estimated by GWAS models were used to estimate pleiotropic effects. Approximately 0.61% (122,000) markers exhibited significant pleiotropic effects across the genome (**Supplementary Figure S5**; **Supplementary Table S6**). Significant associations with pleiotropic effects were identified on all ten sorghum chromosomes, with several novel genetic associations for biomass and its compositional traits identified in addition to well-known genetic loci (*Y1*, *Dw1*, *Dw2*, and *Ma6*). A highly significant locus with pleiotropic effect was identified on Chr5 (~52 Mb) for FW and DW. Another genomic region on Chr6 (~43 Mb) showed a shared association with PH, DTH, ADF, and NFC. Similarly, a genomic region on Chr9 (~57 Mb) exhibited a shared association with PH, DTH, and NDF.

## Discussion

### Population structure and divergence

BAP lines (biomass and sweet sorghum) are usually tall, produce high biomass, and flower later than other sorghum lines due to a significant proportion of photoperiod sensitivity (Brenton et al., 2016). Limited efforts have been made in the genetic and phenotypic characterization of available NPGS collection of bioenergy accessions, particularly for bioenergy-related traits. The BAP is among the most essential genetic resources that now possesses WGS data to advance the breeding of bioenergy sorghums. Characterizing and identifying suitable germplasm lines (*i.e.*, high-biomass, sweet, forage, and grain) will expedite the developmental process of new hybrids and cultivars carrying superior bioenergy-related traits. In this study, we processed the BAP WGS data for the high-throughput assessment of genetic diversity and marker-trait associations underlying complex traits related to biomass yield and vegetative composition. In addition, we used an adaptive shrinkage analysis and identified several genomic regions associated with significant effects on multiple phenotypic traits related to biomass yield and biomass composition in the BAP, which supports the hypothesis that several traits are influenced by the pleiotropic effects of a few major loci.

ADMIXTURE analysis has been widely applied earlier to assess the population structure using diverse panels of sorghum, including the SAP (Casa et al., 2008; Brown et al., 2011; Boatwright et al., 2022a) as well as the BAP (Brenton et al., 2016). Consistent with our earlier observation based on the population structure of the BAP (Brenton et al., 2016), we recognized six groups (*K* = 6) in the ADMIXTURE analysis, including three straightforward sorghum races (kafir, guinea, and caudatum), though the fourth group comprised of guinea-caudatum. As Ethiopia is considered a probable center of origin and diversity for sorghum, the fifth and sixth groups consisted of Ethiopian-durra and Ethiopian-mixed, respectively. The bicolor race represents a minor group in the BAP, and it was considered an early domesticated race that was not separated as an independent group (Harlan and Stemler, 1976; Brown et al., 2011; Wang et al., 2013; Sapkota et al., 2020; Boatwright et al., 2022a). As we know, the LD patterns are critical for designing association mapping experiments and preparing breeding strategies (Flint-Garcia et al., 2003). The whole-genome average LD decay distance was approximately 40 kb (*r^2^* < 0.2), though it varied across the chromosomes (Hamblin et al., 2004; Boatwright et al., 2022a). We observed a slightly higher LD for chromosome 6 of the BAP, consistent with the previous results that found limited recombination on Chr6 (Wang et al., 2013; Hu et al., 2019; Boatwright et al., 2022a).

### Genetic associations for biomass-related traits

Plant height (PH) is an integral part of plant architecture that significantly correlates with biomass production in bioenergy sorghum (Calviño and Messing, 2012; Brenton et al., 2016; Guden et al., 2023). PH is genetically controlled by multiple genes in sorghum, including three predominant loci (*Dw1*: Sobic.009G229800, *Dw2*: Sobic.006G067700, and *Dw3*: Sobic.007G163800). PH showed a highly positive correlation with DTH, FW, DW, and lignin in BAP (Brenton et al., 2016; **Supplementary Table S1**). This study confirmed two dwarfing genes (*Dw1* and *Dw2*) controlling plant height. The genomic region on Chr6 (~42-43 Mb) showed shared associations with other traits (SC, DTH, DW, BRX, ADF, and NFC). However, no QTL appeared on Chr7 or near the location of another dwarfing locus (*Dw3*). The additional shared associations were observed on Chr8 within a 0.7 Mb region for PH and FW and on Chr9 (57-59 Mb) for DTH, FW, and NDF, which were not detected in a previous study using the same panel (Brenton et al., 2016). Another significant locus at Chr10 (1.8 Mb) may correspond to the *waxy* (Sobic.010G022600) locus, which encodes a glycosyl-transferase orthologous to Arabidopsis *granule-bound starch synthase 1* (Boatwright et al., 2022a). The genetic loci associated with plant height and biomass have been previously co-localized (Brown et al., 2008; Boatwright et al., 2022b). Plant breeders strategically target taller genotypes for biomass improvement (Guden et al., 2023). Approximately 60% of the BAP accessions are photoperiod-sensitive; therefore, data scoring on physiological maturity involving a whole set of lines was impossible. However, phenotypic observations were recorded on days to harvest (DTH), representing biomass maturity (Brenton et al., 2016). In addition to the two shared associations between DTH and PH on Chr6 and Chr9, a significant locus was identified on Chr6 (1.8 Mb) close to a known maturity (*Ma6*) locus, which has been previously reported (Mace and Jordan, 2010).

In bioenergy sorghum, overall biomass yield (fresh and dry weight) is influenced by several growth and developmental parameters such as flowering duration, plant height, stem diameter, juice, and lignin content, in addition to the environmental factors, and thus are considered complex traits. However, the previous study did not emphasize the genetic characterization of FW and DW (Brenton et al., 2016). Two of the 11 loci identified in this study on Chr1 (65 Mb) and Chr6 (48 Mb) were overlapped with loci previously reported (Mace et al., 2013; Boatwright et al., 2022b; Souza et al., 2023). Similarly, additional loci identified on the Chr5 for FW were also reported earlier in the overlapping regions (Fiedler et al., 2014), and Chr9 (Mocoeur et al., 2015; Wang et al., 2016). However, the rest of the genomic regions identified in this study were considered novel, including Chr1 (32 Mb), Chr2 (77 Mb), Chr7 (1.4 Mb), and Chr8 (0.6 and 40 Mb).

Overall, 43 QTL were identified for DW, spread on nine of the ten sorghum chromosomes with the exclusion of Chr3. Three significant loci identified in our study were previously reported in the overlapping regions on Chr2 (Kapanigowda et al., 2014), Chr4 (Felderhoff et al., 2012), and Chr6 (Ritter et al., 2008) for total dry biomass. Similarly, two genomic regions coincided with Chr5 (11 and 52 Mb) was reported earlier for dry matter growth rate (Fiedler et al., 2014). Additional two genetic loci identified in the current study were also reported in earlier studies in the overlapping regions, each located on the Chr7 (Somegowda et al., 2022) for PH, and another locus on Chr10 (Fiedler et al., 2014) for dry matter growth rate, and sucrose content using a diverse germplasm of sorghum. Several genomic regions associated with DW were novel, located on Chr1, Chr8, and Chr9. Brix is commonly used in bioenergy sorghum as a reliable indicator of sugar content (Murray et al., 2008; Kawahigashi et al., 2013). For BRX, GWAS identified only three associations in this study, each on Chr3, Chr6, and Chr8. Previous studies overlapped two loci (Chr3 and Chr6) (Souza et al., 2023). A locus on Chr8 (~62 Mb) was identified for the first time and thus considered novel. The QTL identified on Chr6 was also colocalized with a known dwarfing locus (*Dw2*) in sorghum (Higgins et al., 2014; Burrell et al., 2015; Boatwright et al., 2022a) because maturity significantly impacts the brix (%) due to sugar remobilization during the shift from vegetative stage to reproductive.

### Genetic associations for structural and non-structural carbohydrates

Overall, GWAS identified several genomic regions strongly associated with phenotypic traits related to biomass composition, including structural (ADF and NDF) and non-structural carbohydrates (NFC). The genomic loci associated with ADF identified on Chr3, Chr6, and Chr8 overlapped with a previous study conducted by Boatwright et al. (2022b) using the CP-NAM population, which was developed especially for carbon-partitioning traits from the cross between the parental lines selected from the BAP. A highly significant locus identified on Chr6 (~43 Mb) was co-localized with the plant height locus *Dw2* locus. This is unsurprising because ADF showed a positive relationship with PH in the BAP. The remaining two associations, including two loci on Chr1 and a locus on Chr10, were detected for the first time and thus considered novel (**Supplementary Table S4**).

NDF is a major component of the biomass composition and plays a significant role in forage quality. Based on the LD decay observations, the most significant associations (67 loci) were identified for NDF (**Supplementary Figure S1**; **Table 1**), including some candidate genes described in the results section. A locus was detected on Chr1 at 24 Mb, which overlapped with an earlier study (Boatwright et al., 2022b). Genetic loci located on Chr2 (~9 Mb), Chr9 (~58 Mb), and Chr10 (~10 Mb) overlapped with previous studies (Li et al., 2018; Boatwright et al., 2022). Similarly, a highly significant locus detected on Chr6 (~60 Mb) overlapped with an earlier study (Shiringani and Friedt, 2011). However, a locus detected in this study on Chr5 (~5 Mb) was associated with bioenergy-related traits like stem hydrolysis yield potential and stem sugar release (Van der Auwera et al., 2013).

Several significant loci were identified for NFC in the present study, corroborated with earlier studies for various biomass-related traits, days to flowering, plant height, tiller number, shoot cylinder height, cellulose content, and hemicellulose content. A genomic region associated with NFC on Chr6 (43 Mb) was overlapped in previous studies with various traits, including brix %, days to flowering, and total dry biomass (Ritter et al., 2008; Felderhoff et al., 2012), shoot dry biomass (Zhang et al., 2015), and plant height (Burrell et al., 2015; Gelli et al., 2016; McCormick et al., 2018). Another genomic region on Chr6 (Chr06: 48,838,810) overlapped with the locus associated with juice yield (Mace and Jordan, 2010; Mocoeur et al., 2015) and fresh biomass (Wang et al., 2014). There were multiple genomic regions significantly associated with NFC on Chr8 (5-6 Mb) were overlapped in previous studies for other traits, including the NDF (Li et al., 2018), cellulose, hemicellulose content (Murray et al., 2008), stem circumference (Zhao et al., 2016), and plant height (Girma et al., 2019).

## Conclusion

We genetically characterized a bioenergy association panel based on whole-genome sequencing data. Significant nucleotide diversity and heterozygosity were observed between the photoperiod-sensitive and insensitive individuals of the panel. Six genomic regions showed strong selection sweeps on different chromosomes (Chr1, Chr2, Chr3, Chr5, Chr6, and Chr8) based on the *F_st_* estimates. In addition, we used a set of high-quality SNP markers (~ 5.48 million) for genome-wide marker-trait associations for various traits related to biomass (DTH, PH, FW, and DW) and its composition (ADF, NDF, NFC, and lignin). For FW and DW, several significant genomic regions were identified on the Chr1, Chr2, Chr 5, Chr 6, and Chr8. Similarly, highly significant genomic regions were identified on the Chr1, Chr3, Chr5, Chr6, and Chr8 for biomass compositional traits (ADF, NDF and NFC). We also identified several significant genomic loci with pleiotropic effects across the genome in addition to some well-characterized genes for plant height (*Dw1* and *Dw2*) and the *YELLOW SEED1* locus (*Y1*) for seed color. Identified marker-trait associations can be used to select superior parental lines for developing mapping populations for high-resolution mapping studies for a specific set of bioenergy-related traits. In addition, we identified several significant SNPs corresponding to the putative candidate genes that can be used for functional characterization using genome-editing technology to know their precise role in regulating specific bioenergy-related traits.

## Data availability statement

The original contributions presented in the study are publicly available. The SNP data for BAP is available from the European Variant Archive, accession number PRJEB72639.

## Author contributions

NK: Conceptualization, Data curation, Formal Analysis, Investigation, Methodology, Validation, Writing – original draft, Writing – review & editing. JB: Conceptualization, Data curation, Formal Analysis, Investigation, Methodology, Software, Validation, Writing – review & editing. RB: Conceptualization, Data curation, Validation, Writing – review & editing. ZB: Data curation, Formal Analysis, Investigation, Supervision, Writing – review & editing. SK: Conceptualization, Data curation, Funding acquisition, Project administration, Resources, Supervision, Validation, Writing – review & editing.
