# Supplementary material for: Identification of pleiotropic loci mediating structural and non-structural carbohydrate accumulation within the sorghum bioenergy association panel using high-throughput markers
Source: Front Plant Sci. 2024 Feb 28;15:1356619. doi: 10.3389/fpls.2024.1356619 (PMC10933071; doi:10.3389/fpls.2024.1356619)
Supplement: Supplementary file 1 [file DataSheet_1.pdf]

Supplemental Figures:

Identification of pleiotropic loci mediating structural and non-structural carbohydrate accumulation within the sorghum bioenergy association panel using high-throughput markers

Neeraj Kumar[1,2,†,*], J. Lucas Boatwright[1,2,†,*], Richard E. Boyles[2,3], Zachary W. Brenton[1,4], Stephen Kresovich[1,2,5]

[1]Advanced Plant Technology, Clemson University, Clemson, SC 29634, [2]Department of Plant and Environmental Sciences, Clemson University, Clemson, SC 29634, [2,3]Pee Dee Research and Education Center, Clemson University, Florence, SC 29506, [4]Carolina Seed Systems, Darlington, SC 29532, [5]Feed the Future Innovation Lab for Crop Improvement, Cornell University, Ithaca, NY, USA, [†]These authors contributed equally to this work, [*]Corresponding authors.

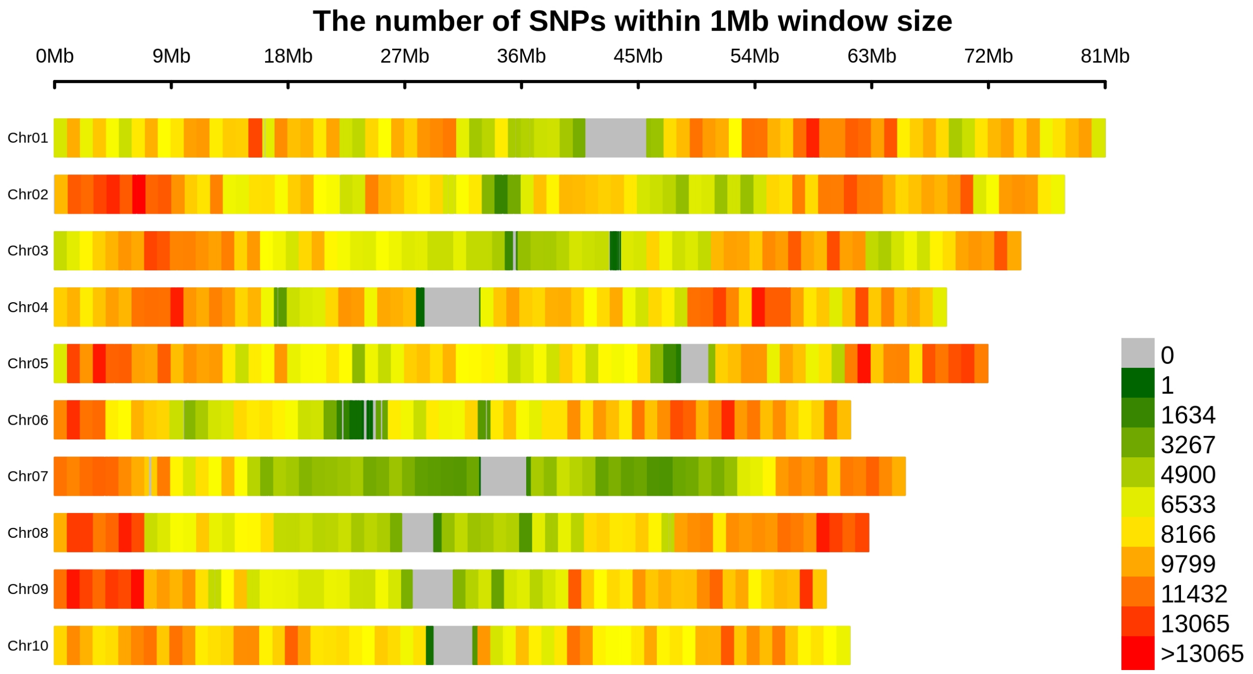

Supplementary Figure S1: Single nucleotide polymorphism (SNP) density on each chromosome of the bioenergy association panel (BAP).

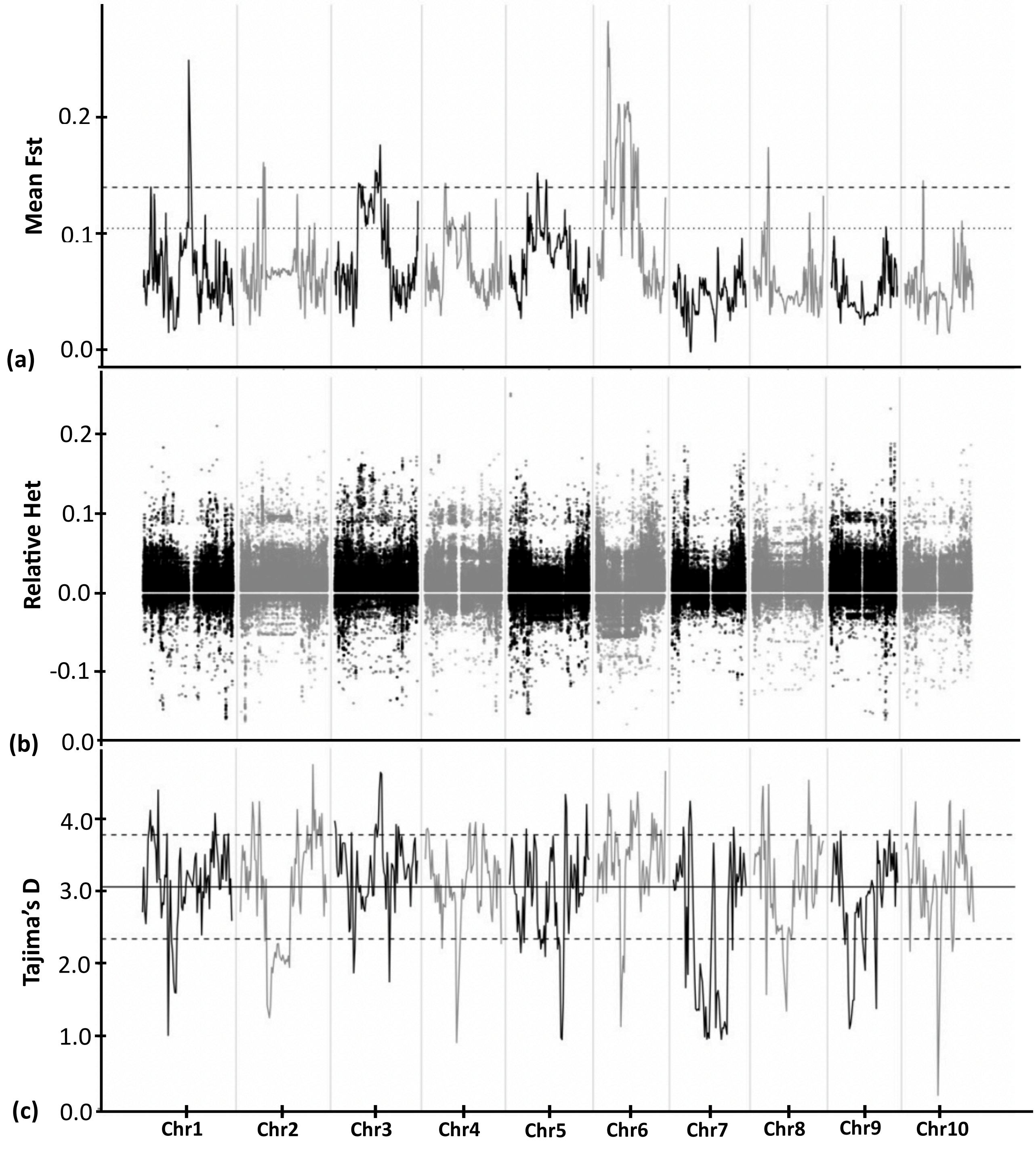

Supplementary Figure S2. Genome-wide signatures of selection. (a) The mean F_{st} across the sorghum genome between photoperiod sensitive and insensitive accessions. (b) Expected heterozygosity in the photoperiod sensitive and insensitive group. (c) Genome-wide Tajima's D estimates. The horizontal lines show the mean and standard deviations of the estimates; solid black lines show the genome-wide average Tajima's D; dotted lines show the mean plus 1 standard deviation (SD); and long dashed lines show the mean plus 2 SD of the estimates. Vertical dotted lines show genes and loci related to height, maturity, and other domestication-related traits.

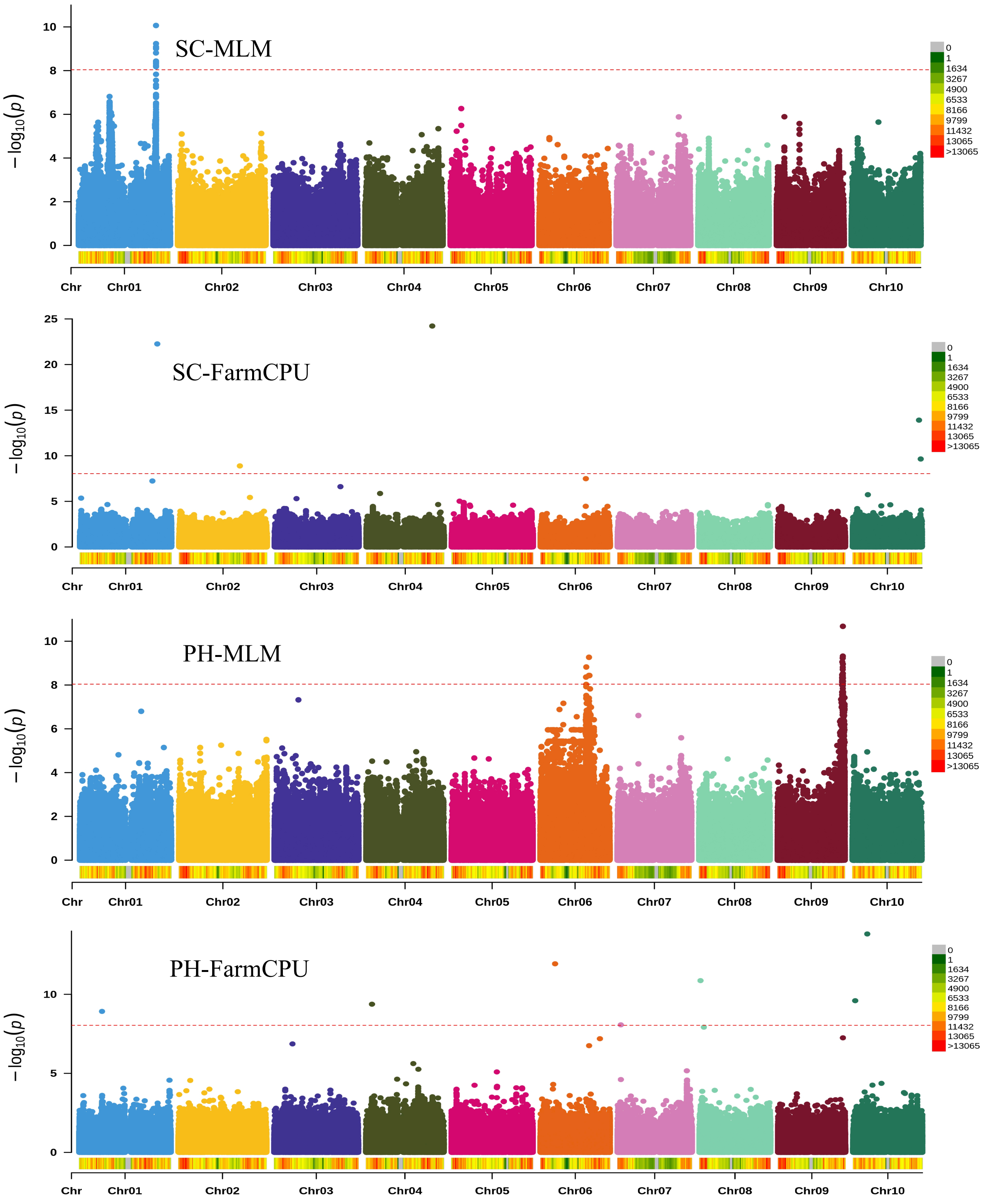

SC-MLM
$-\log_{10}(p)$
0
2
4
6
8
10
Chr
Chr01
Chr02
Chr03
Chr04
Chr05
Chr06
Chr07
Chr08
Chr09
Chr10
0
1
1634
3267
4900
6533
8166
9799
11432
13065
>13065
SC-FarmCPU
$-\log_{10}(p)$
0
5
10
15
20
25
Chr
Chr01
Chr02
Chr03
Chr04
Chr05
Chr06
Chr07
Chr08
Chr09
Chr10
0
1
1634
3267
4900
6533
8166
9799
11432
13065
>13065
PH-MLM
$-\log_{10}(p)$
0
2
4
6
8
10
Chr
Chr01
Chr02
Chr03
Chr04
Chr05
Chr06
Chr07
Chr08
Chr09
Chr10
0
1
1634
3267
4900
6533
8166
9799
11432
13065
>13065
PH-FarmCPU
$-\log_{10}(p)$
0
5
10
Chr
Chr01
Chr02
Chr03
Chr04
Chr05
Chr06
Chr07
Chr08
Chr09
Chr10
0
1
1634
3267
4900
6533
8166
9799
11432
13065
>13065

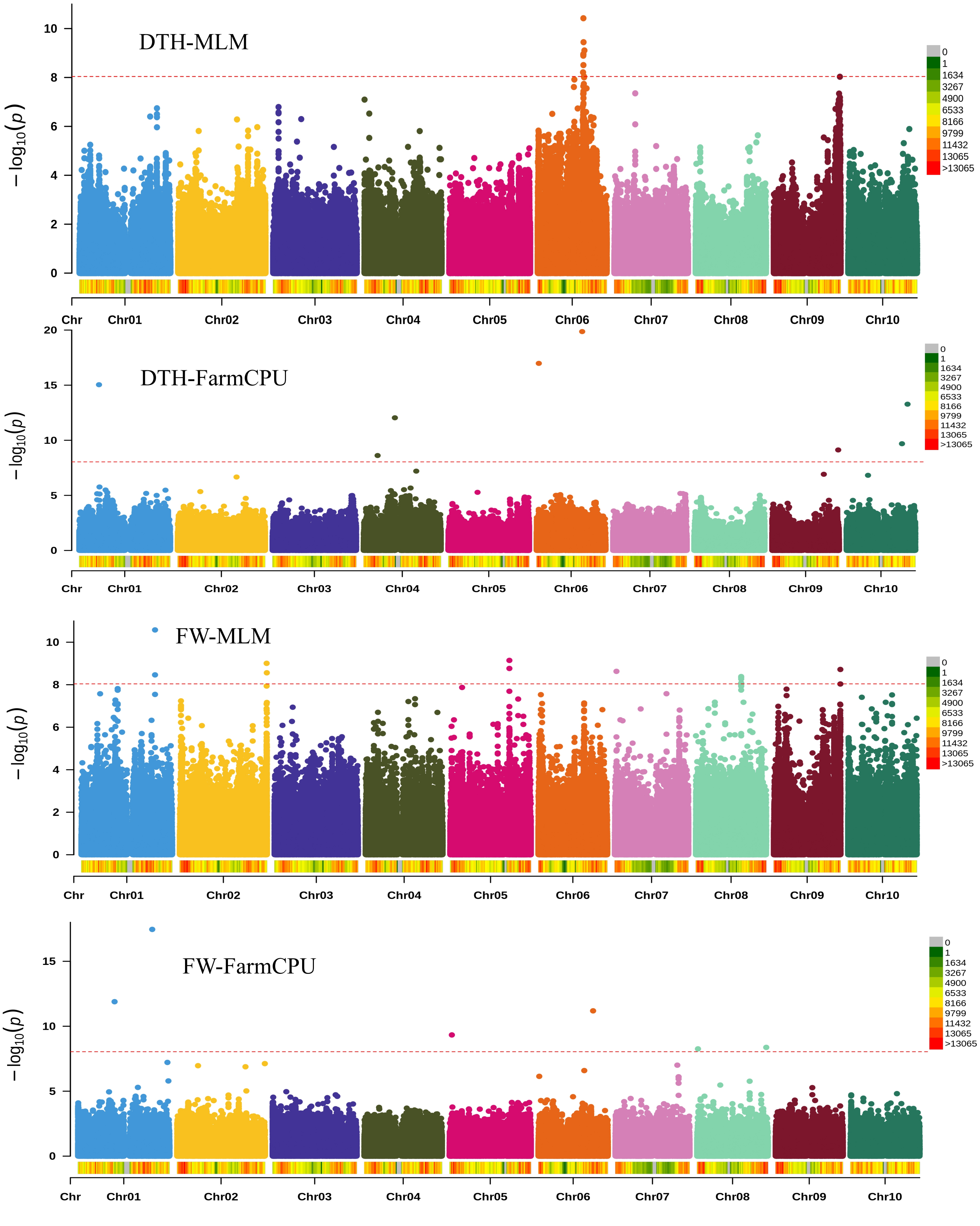

DTH-MLM
DTH-FarmCPU
FW-MLM
FW-FarmCPU
$-\log_{10}(p)$
Chr
Chr01
Chr02
Chr03
Chr04
Chr05
Chr06
Chr07
Chr08
Chr09
Chr10
0
1
1634
3267
4900
6533
8166
9799
11432
13065
>13065

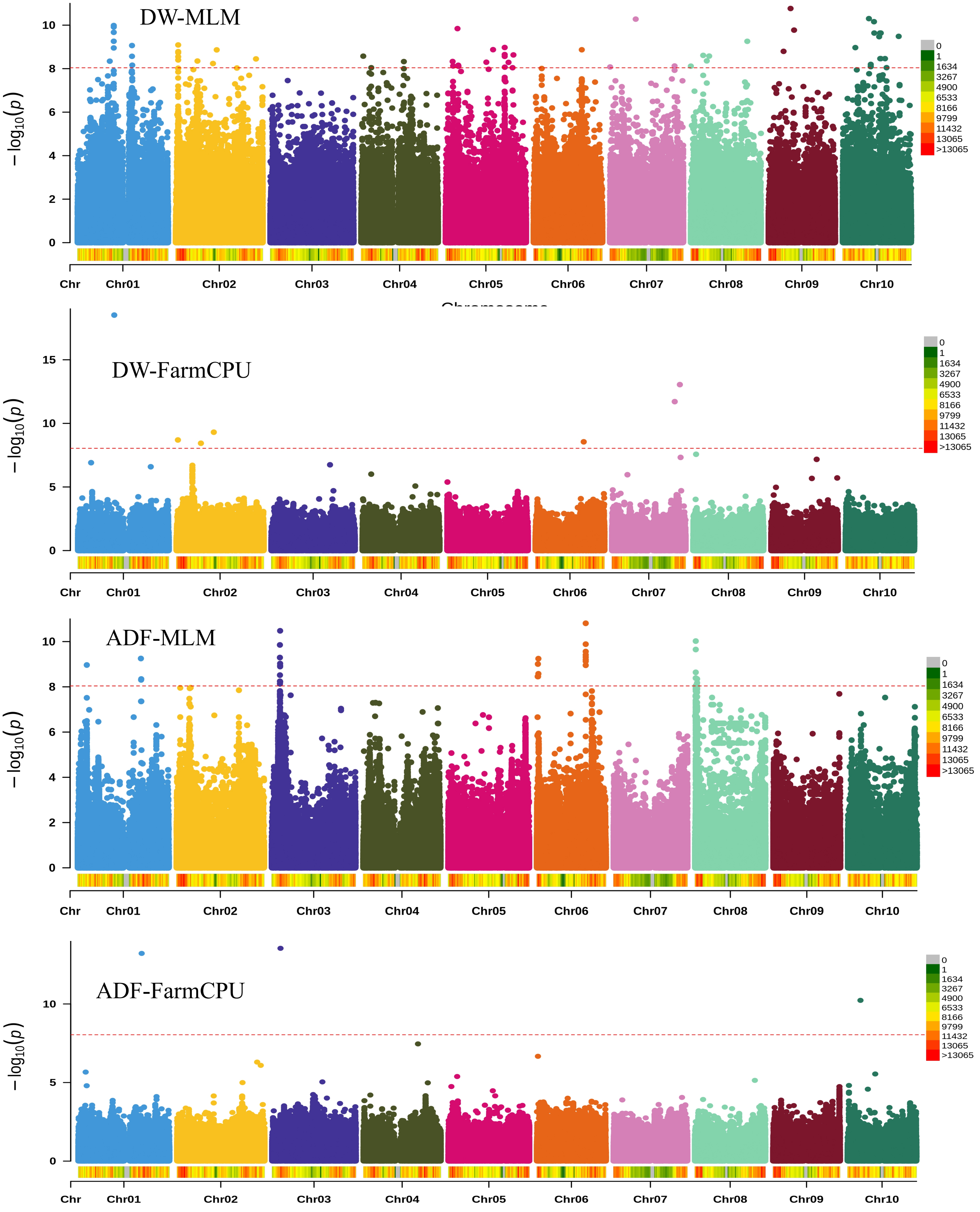
DW-MLM
DW-FarmCPU
ADF-MLM
ADF-FarmCPU
$-\log_{10}(p)$
Chr
Chr01
Chr02
Chr03
Chr04
Chr05
Chr06
Chr07
Chr08
Chr09
Chr10
0
1
1634
3267
4900
6533
8166
9799
11432
13065
>13065

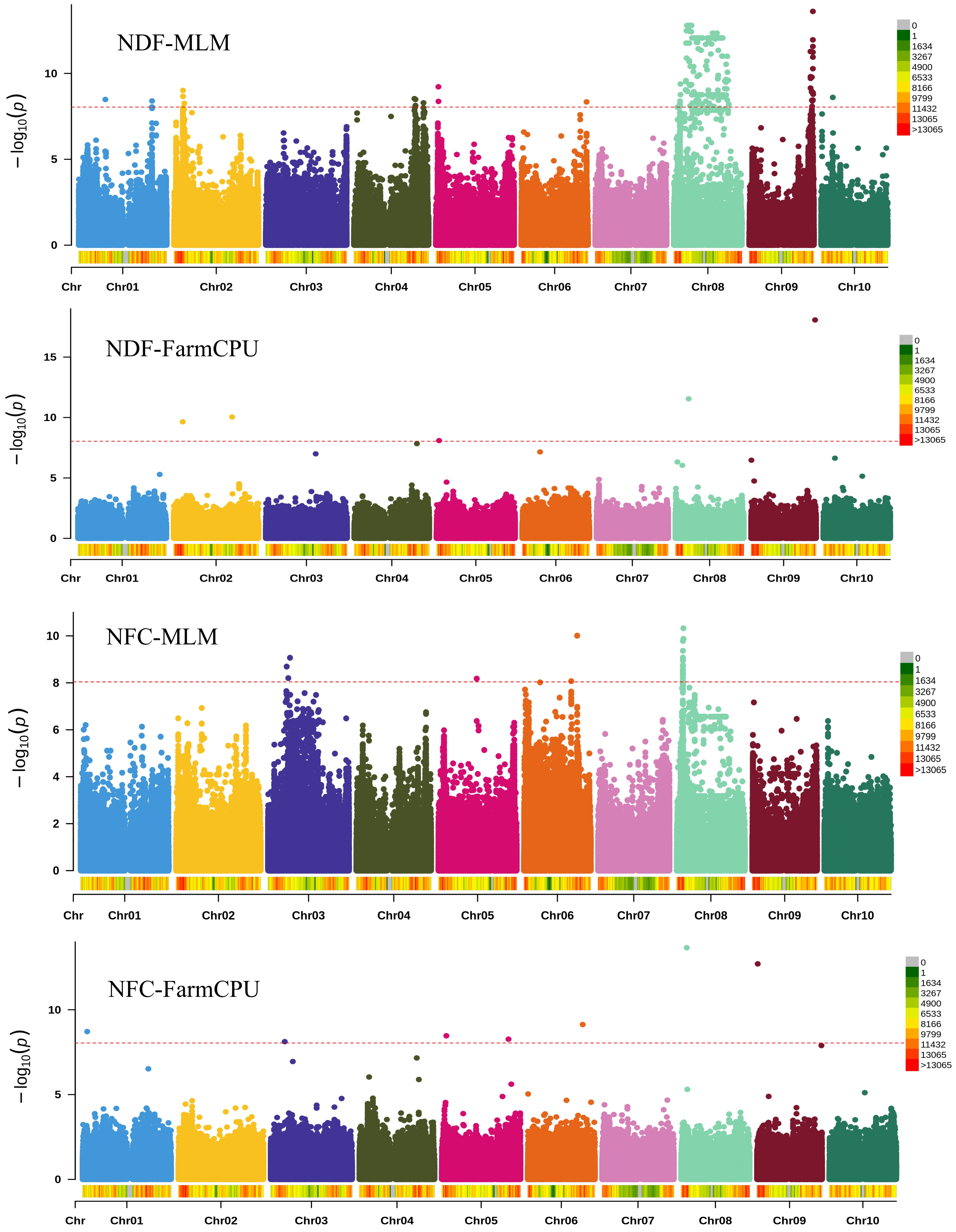
NDF-MLM
NDF-FarmCPU
NFC-MLM
NFC-FarmCPU
$-\log_{10}(p)$
0
5
10
15
0
2
4
6
8
10
Chr
Chr01
Chr02
Chr03
Chr04
Chr05
Chr06
Chr07
Chr08
Chr09
Chr10
0
1
1634
3267
4900
6533
8166
9799
11432
13065
>13065

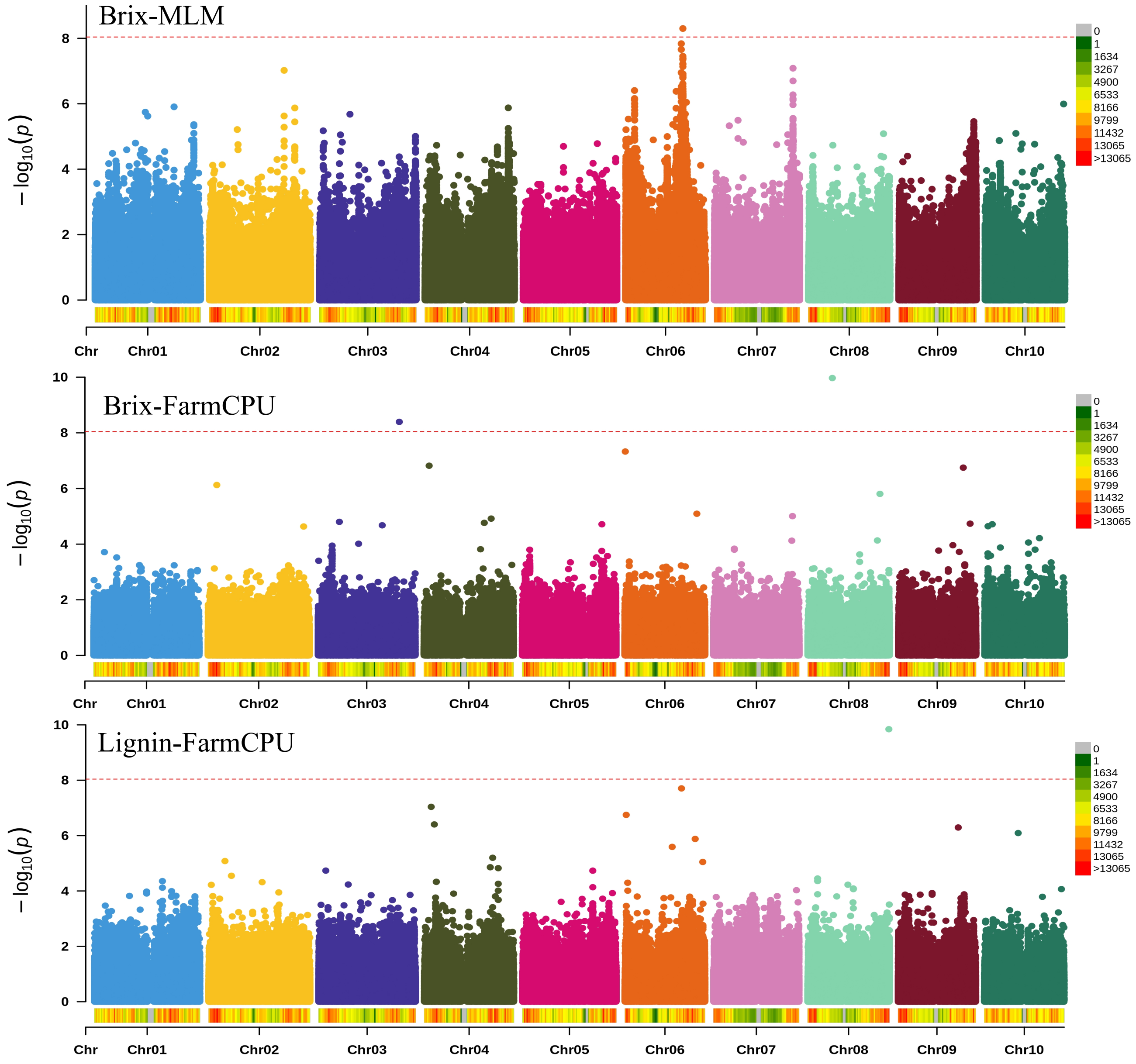

Supplementary Figure S3. Manhattan plots of genome-wide association for various traits using two models (MLM and FarmCPU). (A) Seed color (SC), plant height (PH), days to harvest (DTH), fresh weight (FW), and dry weight (DW). (B) acid detergent fiber (ADF), neutral detergent fiber (NDF), non-fibrous carbohydrate (NFC), Brix, and lignin. The $-\log_{10}(p)$ values (y-axis) are plotted against the position on each chromosome (x-axis). Each solid circle represents a SNP, and the red dashed line represents the Bonferroni-corrected threshold ($p \leq 0.05$).

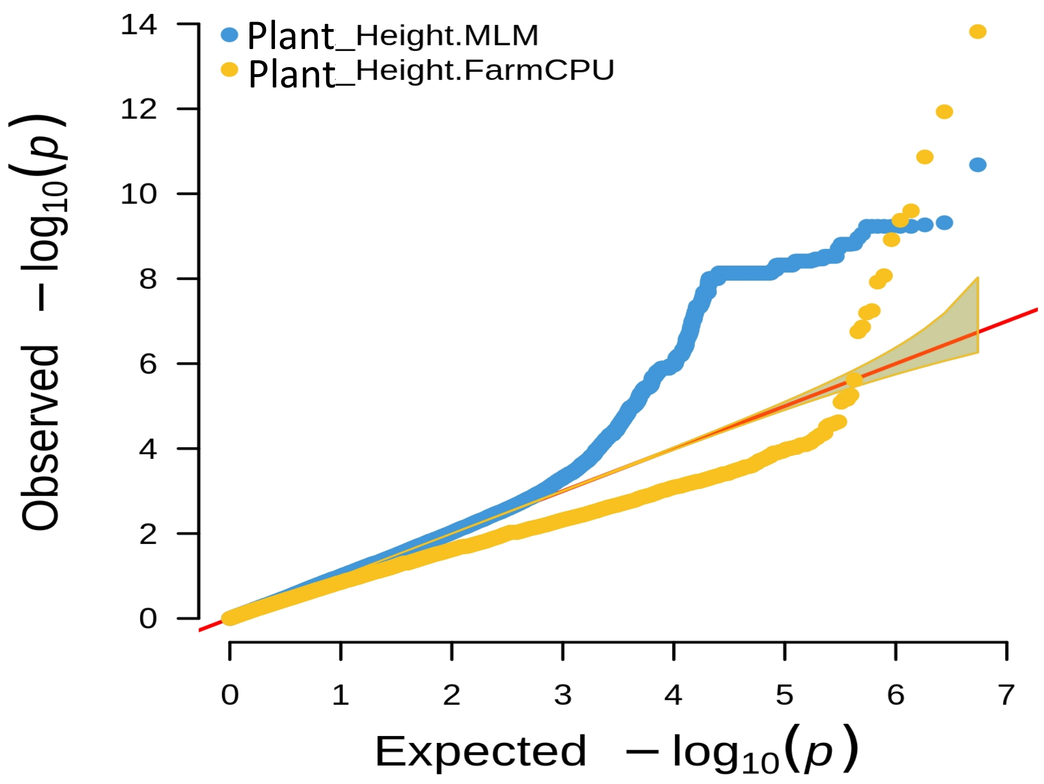
Plant_Height.MLM
Plant_Height.FarmCPU
Observed −log10(p)
Expected −log10(p)
0
2
4
6
8
10
12
14
0
1
2
3
4
5
6
7

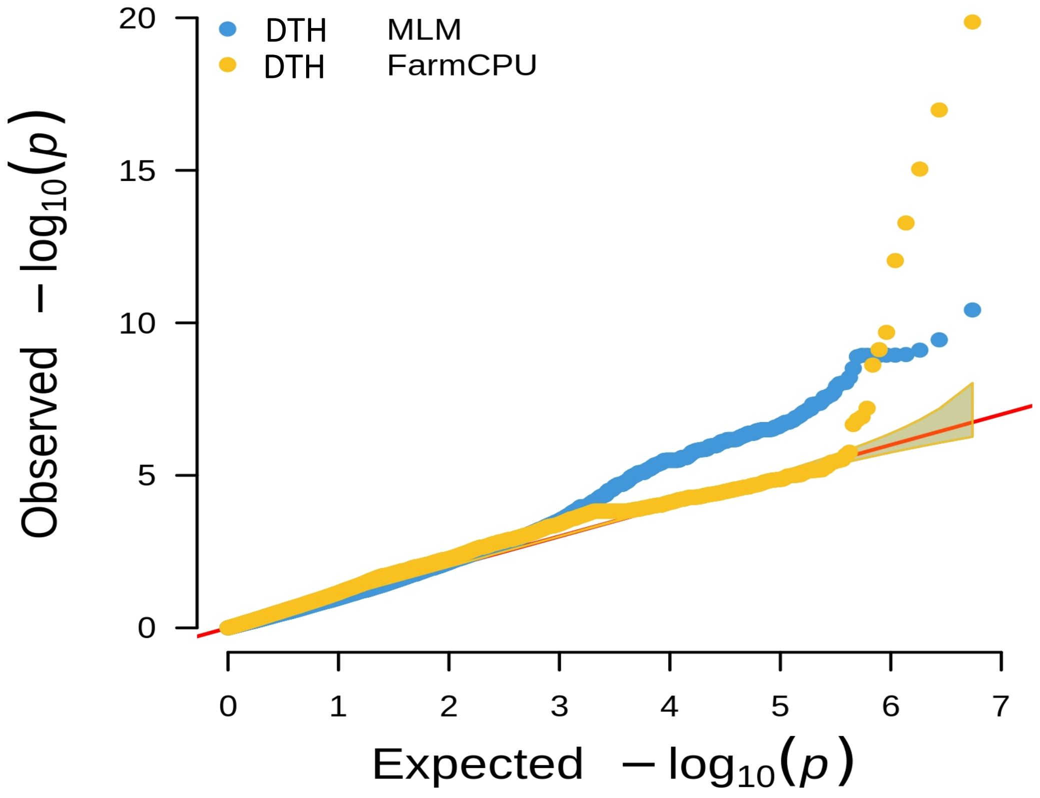
DTH MLM
DTH FarmCPU
Observed −log10(p)
Expected −log10(p)
0
5
10
15
20
0
1
2
3
4
5
6
7

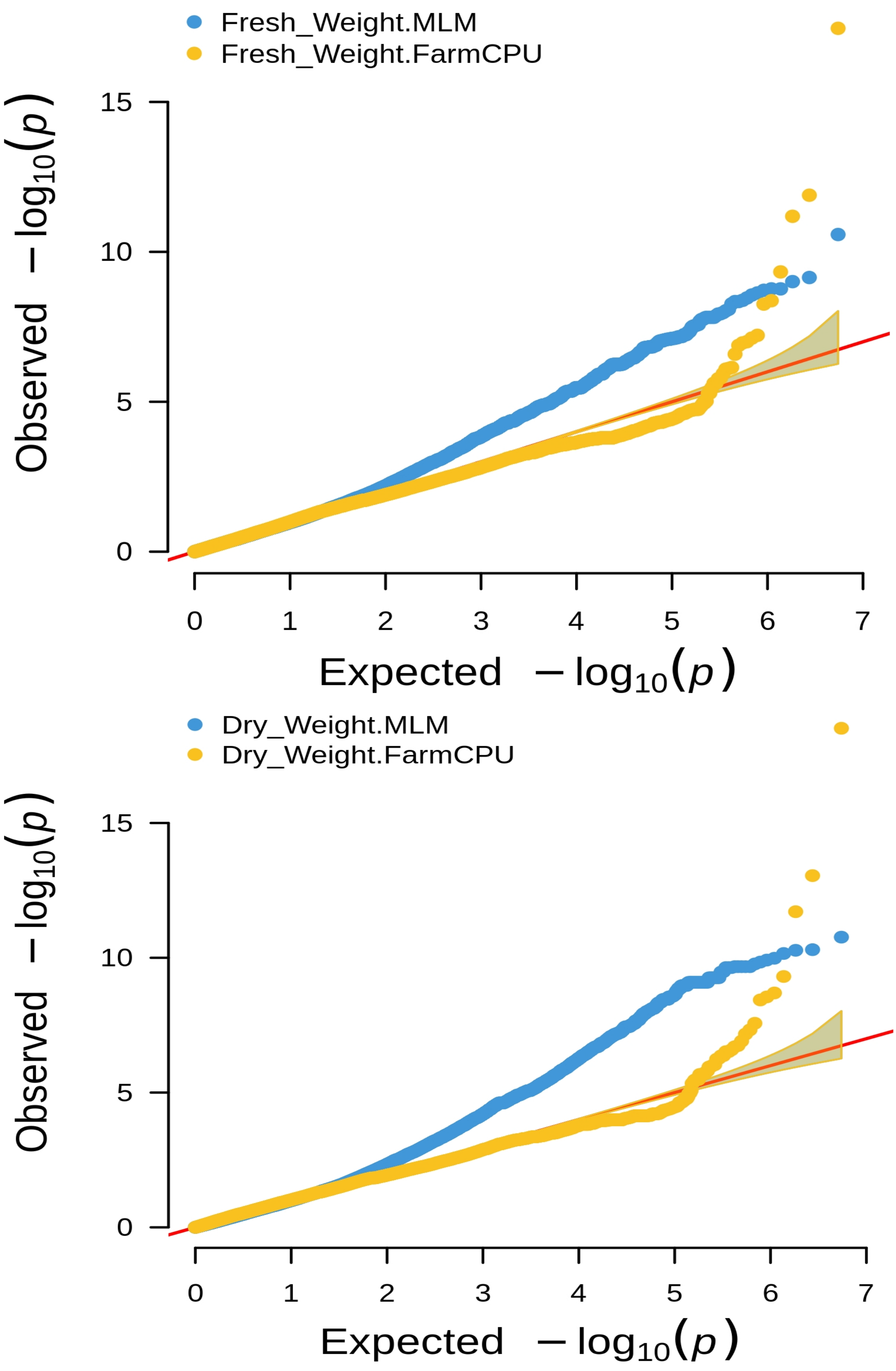
Fresh_Weight.MLM
Fresh_Weight.FarmCPU
Observed − log10(p)
15
10
5
0
0
1
2
3
4
5
6
7
Expected − log10(p)
Dry_Weight.MLM
Dry_Weight.FarmCPU
Observed − log10(p)
15
10
5
0
0
1
2
3
4
5
6
7
Expected − log10(p)

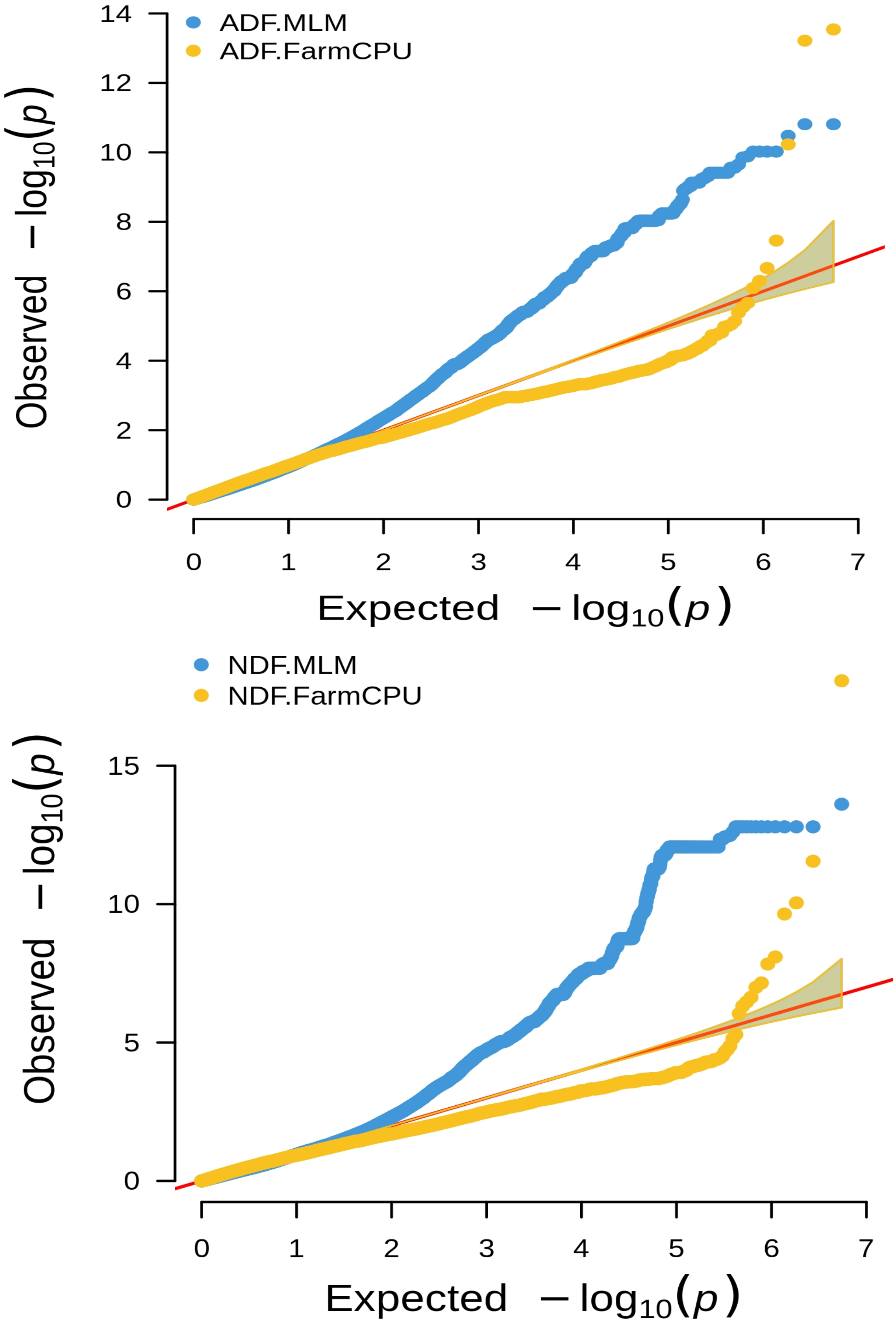

ADF.MLM
ADF.FarmCPU
Observed −log10(p)
Expected −log10(p)
0
2
4
6
8
10
12
14
0
1
2
3
4
5
6
7
NDF.MLM
NDF.FarmCPU
Observed −log10(p)
Expected −log10(p)
0
5
10
15
0
1
2
3
4
5
6
7

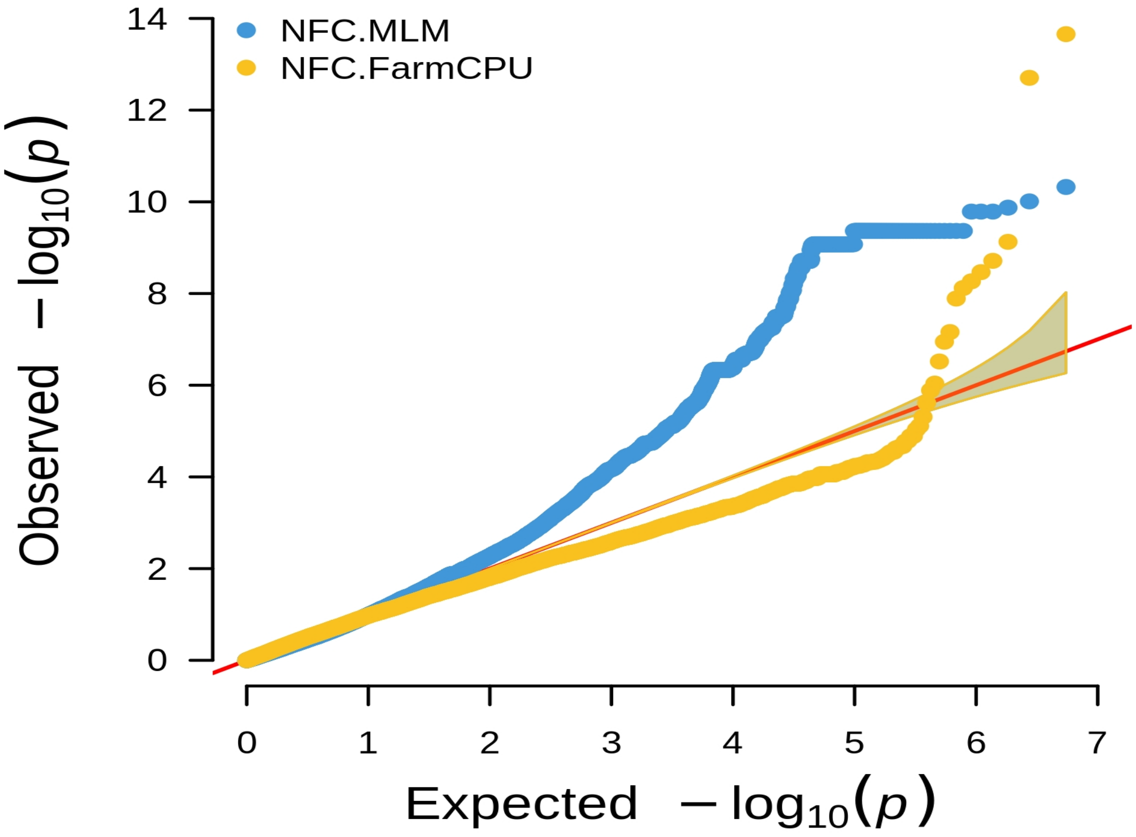
NFC.MLM
NFC.FarmCPU
Observed −log10(p)
Expected −log10(p)
0
2
4
6
8
10
12
14
0
1
2
3
4
5
6
7

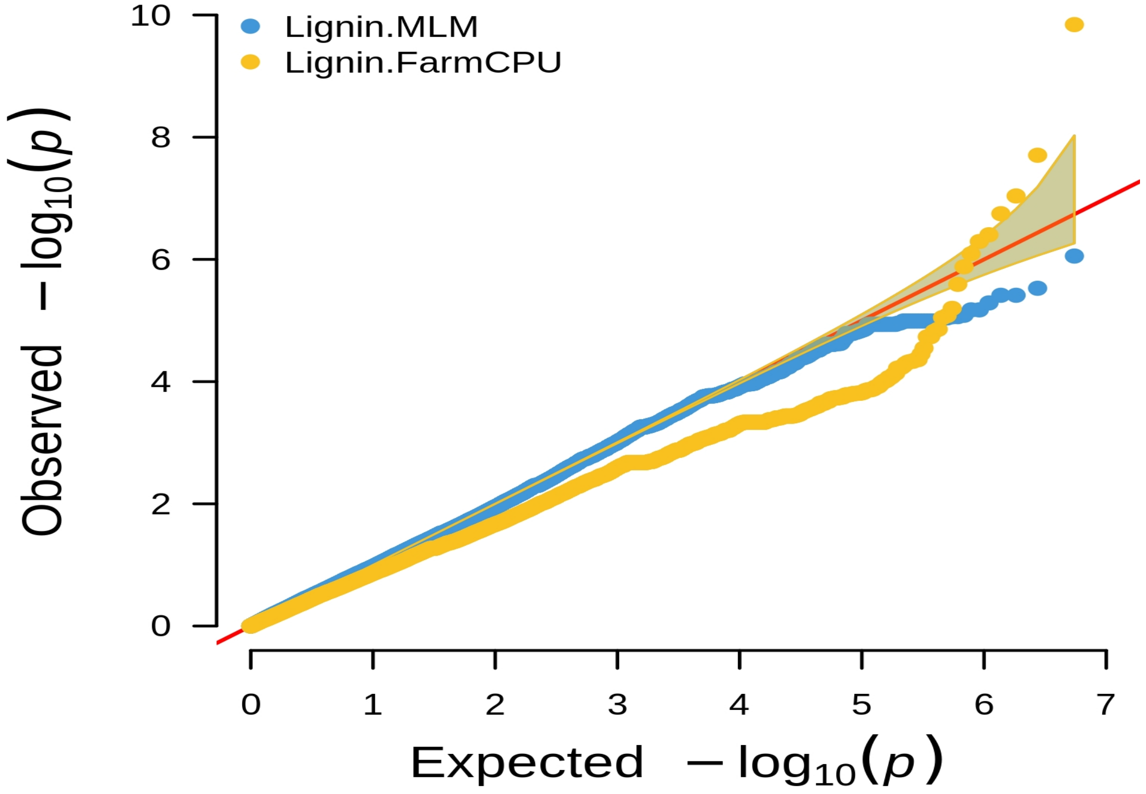
Lignin.MLM
Lignin.FarmCPU
Observed −log10(p)
Expected −log10(p)
0
2
4
6
8
10
0
1
2
3
4
5
6
7

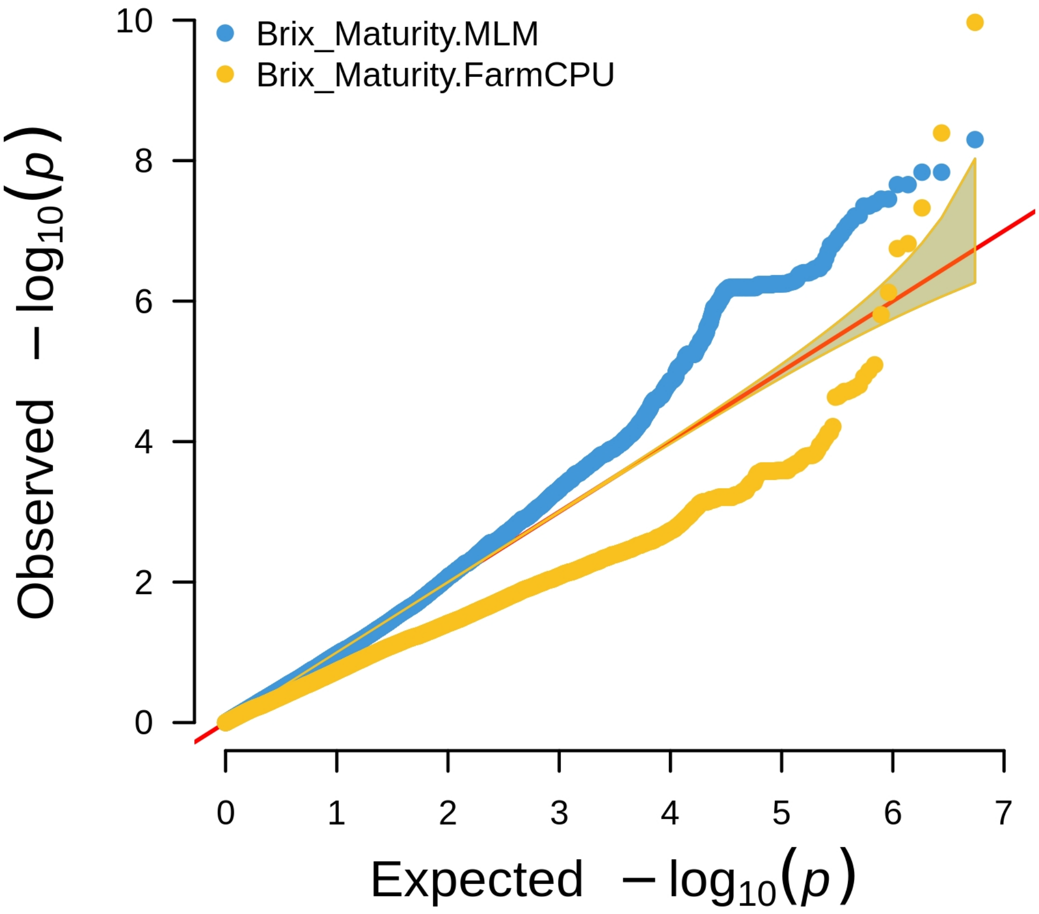

Supplementary Figure S4. Quantile-quantile (QQ) plot of each trait using two models (MLM and FarmCPU) in GWAS analysis of BAP. Each plot represents the expected -log$_{10}$ (*p*) values on the *x*-axis, whereas the observed *p*-values are on the *y*-axis for both these models.

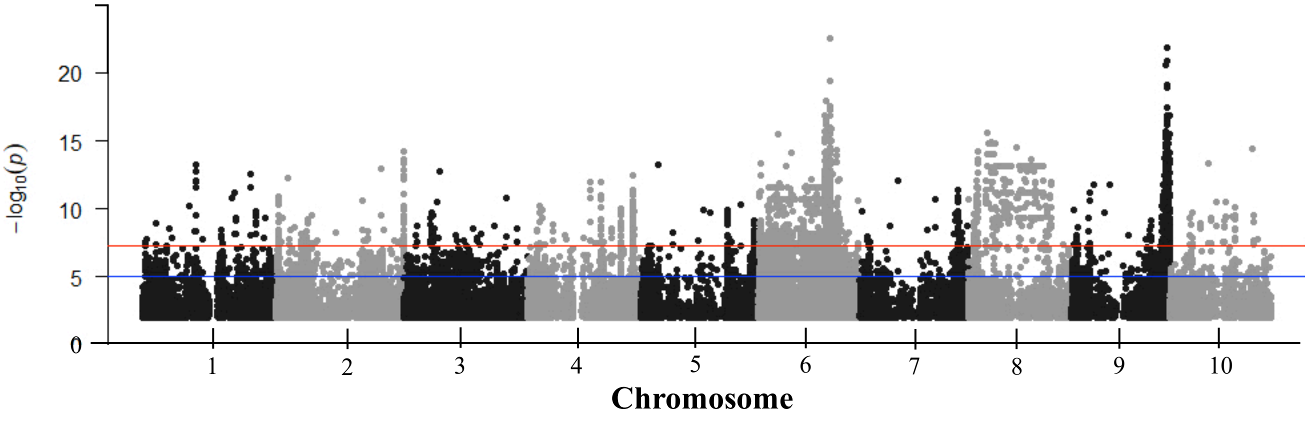

Supplementary Figure S5. Manhattan plots showing genome-wide measures of pleiotropic effects associated regions for various traits of bioenergy association panel. The horizontal blue line represents a Bonferroni-adjusted threshold of 0.05, and the horizontal red line represents a Bonferroni-adjusted threshold of 0.01.
